# SH2B1 Tunes Hippocampal ERK Signaling to Influence Fluid Intelligence in Humans and Mice

**DOI:** 10.34133/research.0269

**Published:** 2023-11-14

**Authors:** Xiujuan Du, Yuhua Yan, Juehua Yu, Tailin Zhu, Chu-Chung Huang, Lingli Zhang, Xingyue Shan, Ren Li, Yuan Dai, Hui Lv, Xiao-Yong Zhang, Jianfeng Feng, Wei-Guang Li, Qiang Luo, Fei Li

**Affiliations:** ^1^ Developmental and Behavioral Pediatric Department, Brain and Behavioral Research Unit of Shanghai Institute for Pediatric Research and Ministry of Education-Shanghai Key Laboratory for Children’s Environmental Health, Xinhua Hospital Affiliated to Shanghai Jiao Tong University School of Medicine, Shanghai 200092, China.; ^2^ Developmental and Behavioral Pediatric Department, Shanghai Xinhua Children’s Hospital, Shanghai 200092, China.; ^3^ National Clinical Research Center for Aging and Medicine at Huashan Hospital, Institute of Science and Technology for Brain-Inspired Intelligence, Ministry of Education-Key Laboratory of Computational Neuroscience and Brain-Inspired Intelligence, Fudan University, Shanghai 200433, China.; ^4^ State Key Laboratory of Medical Neurobiology and Ministry of Education Frontiers Center for Brain Science, Institutes of Brain Science and Human Phenom Institute, Fudan University, Shanghai 200032, China.; ^5^ Shanghai Key Laboratory of Brain Functional Genomics (Ministry of Education), School of Life Sciences, East China Normal University, Shanghai 200062, China.; ^6^ Department of Rehabilitation Medicine, Huashan Hospital, Institute for Translational Brain Research, State Key Laboratory of Medical Neurobiology and Ministry of Education Frontiers Center for Brain Science, Fudan University, Shanghai 200032, China.; ^7^ Center for Experimental Studies and Research, The First Affiliated Hospital of Kunming Medical University, Kunming 650032, China.; ^8^ Shanghai Research Center for Brain Science and Brain-Inspired Intelligence, Shanghai 201210, China.; ^9^ Institute of Cognitive Neuroscience, School of Psychology and Cognitive Science, East China Normal University, Shanghai 200062, China.

## Abstract

Fluid intelligence is a cognitive domain that encompasses general reasoning, pattern recognition, and problem-solving abilities independent of task-specific experience. Understanding its genetic and neural underpinnings is critical yet challenging for predicting human development, lifelong health, and well-being. One approach to address this challenge is to map the network of correlations between intelligence and other constructs. In the current study, we performed a genome-wide association study using fluid intelligence quotient scores from the UK Biobank to explore the genetic architecture of the associations between obesity risk and fluid intelligence. Our results revealed novel common genetic loci (*SH2B1*, *TUFM*, *ATP2A1*, and *FOXO3*) underlying the association between fluid intelligence and body metabolism. Surprisingly, we demonstrated that *SH2B1* variation influenced fluid intelligence independently of its effects on metabolism but partially mediated its association with bilateral hippocampal volume. Consistently, selective genetic ablation of *Sh2b1* in the mouse hippocampus, particularly in inhibitory neurons, but not in excitatory neurons, significantly impaired working memory, short-term novel object recognition memory, and behavioral flexibility, but not spatial learning and memory, mirroring the human intellectual performance. Single-cell genetic profiling of Sh2B1-regulated molecular pathways revealed that *Sh2b1* deletion resulted in aberrantly enhanced extracellular signal-regulated kinase (ERK) signaling, whereas pharmacological inhibition of ERK signaling reversed the associated behavioral impairment. Our cross-species study thus provides unprecedented insight into the role of *SH2B1* in fluid intelligence and has implications for understanding the genetic and neural underpinnings of lifelong mental health and well-being.

## Introduction

Intelligence represents a substantial and valid dimension of individual variation, encompassing reasoning, problem-solving, abstract thinking, and learning [1]. The impact of intelligence on various socioeconomic, psychological, lifespan, and health outcomes highlights the importance of understanding its genetic and neural underpinnings [2]. Within the domain of intelligence, fluid intelligence (FI) stands out as the capacity for reasoning, pattern recognition, and tackling novel problems independently of task-specific experience. Human FI is known to be heritable and influenced by environmental factors [1,3,4]. Molecular genetic studies have unveiled specific genes associated with brain function that may contribute to FI, including apolipoprotein E [5], catechol-O-methyl transferase [6], and brain-derived neurotrophic factor (*BDNF*) [7]. Moreover, genome-wide association studies (GWASs) have identified multiple genes associated with intelligence [8–10], shedding light on its genetic architecture. Notably, many of these genes are prominently expressed in the brain, contributing to neurodevelopment and synaptic function [9,10]. However, it is noteworthy that some of these genes, such as *ATP2A1*, *COL16A1*, *DCC*, *FOXO3*, *NEGR1*, *SH2B1*, *SKAP1*, *TUFM*, and *YIPF7* [11–16], have also been linked to body mass index (BMI), obesity, or both. The observed overlap between the genetic determinants of intelligence and body metabolism raises intriguing questions regarding their potential associations.

The relationship between intelligence and body metabolism has long been implicated, suggesting that being overweight or obese can have negative effects on cognitive function. Increasing evidence supports the association between high BMI and poorer performance in neuropsychological intelligence tests [17,18]. Moreover, the combination of elevated BMI and waist-to-hip ratio is an important risk factor for gray matter atrophy in key brain regions including the hippocampus, anterior cingulate cortex, and medial prefrontal cortex [19–21], which are known to contribute to intelligence [19,22–24]. Neuroinflammation related to obesity is proposed as a major contributor to structural alterations in the brain, affecting a lot of brain regions including the amygdala, brainstem, cortex, hippocampus, and hypothalamus, thereby impairing cerebral function [25]. The effects of insulin resistance on both the brain and systemic metabolic organs are characteristic features of metabolic and cognitive dysfunction [26–28], and they may mediate the impact of overweight or obese on intelligence. Alternatively, the associations observed between intelligence and body metabolism may indicate that reduced cognitive ability has a genetic basis, simultaneously affecting metabolic and cognitive processes. Therefore, the identification of genes with polymorphisms that influence intelligence independently of metabolism could help distinguish whether reduced cognitive ability and obesity are parallel phenomena stemming from coincidental genetic factors or interconnected outcomes driven by complex pathophysiological cycles.

**Fig. 1.**
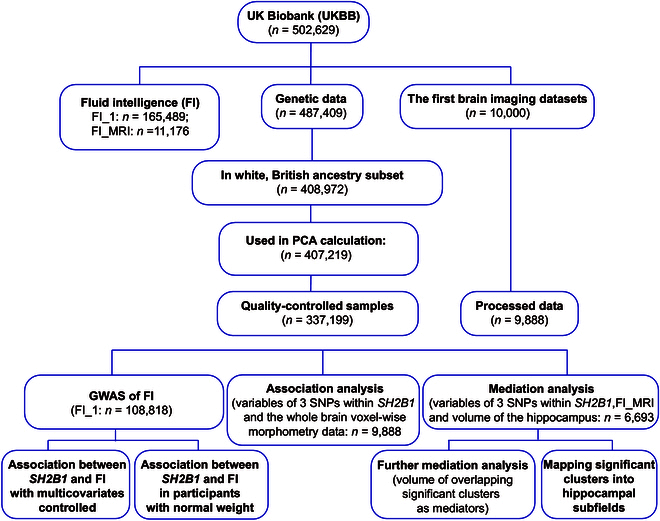
Selection of the UKBB cohort for a study on identification of SH2B1 in fluid intelligence. Participants with all cognitive tests, genetic data, and neuroimaging data available entered the main analyses. PCA, principal component analysis; MRI, magnetic resonance imaging.

In this study, we leveraged the UK Biobank (UKBB), a large-scale population-based cohort comprising approximately 500,000 individuals, to conduct GWASs aimed at identifying candidate genes associated with both obesity risk and FI. Additionally, we employed voxel-based association analysis and mediation analysis to explore whether these associations are influenced by structural changes in specific brain regions (Fig. 1). To establish a causal link with intelligence, we employed conditional knockout mice to examine the brain-region-specific behavioral functions of the newly identified candidate genes. Our objective was to present a conceptual framework that elucidates the genetic and neural connections between FI and body metabolism.

## Results

### GWAS analysis revealed *SH2B1* as a genetic correlate connecting FI and metabolism in humans

**Fig. 2.**
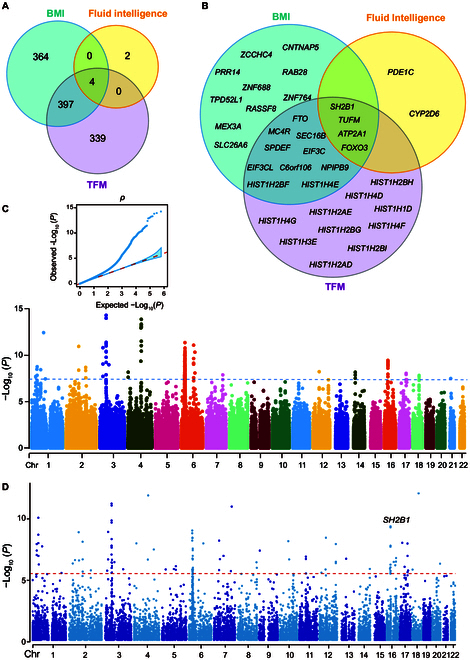
Genome-wide genetic association of FI. (A) Venn diagram showing the numbers of overlapping genes associated with TFM, BMI, and FI. (B) Venn diagram visualizing the intersections between the gene lists, showing only the top 10 genes. (C and D) The results of the GWAS. (C) An SNP-based GWAS Manhattan plot of FI, with age at baseline, sex, BMI at baseline, and 15 PCs adjusted for in the linear model. The negative log10-transformed *P* value (*y*-axis) for each SNP is plotted against the chromosomal position (*x*-axis). The blue line represents the genome-wide significance level at *P* = 5.0E−8. (D) Gene-based GWAS Manhattan plot. The red dotted line represents genome-wide significance (generated by MAGMA).

We performed a GWAS examining the associations between FI, BMI, and trunk fat mass (TFM) using the PhenoScanner online resource, which consolidates from large-scale GWASs [29]. Among the 4 genes displaying significant GWAS signals (*P <* 5.0E−10; Tables S1 to S3) for FI, BMI, and TFM, SH2B adaptor protein 1 (*SH2B1*; NCBI Gene 25970) exhibited the strongest association with both BMI and TFM. Other genes showing significant associations were Tu translation elongation factor, mitochondrial (*TUFM*; NCBI Gene 7284), ATPase sarcoplasmic/endoplasmic reticulum Ca^2+^ transporting 1 (*ATP2A1*; NCBI Gene 487), and forkhead box O3 (*FOXO3*; NCBI Gene 2309) (Fig. 2A and B).

*SH2B1*, which belongs to the family of SH2-domain containing mediators [30], has previously been associated with severe early-onset obesity [31,32] and has been shown to influence human intelligence [8]. To examine the genetic associations between intelligence and metabolism-related traits, we conducted a GWAS of intelligence in the UKBB cohort, controlling for BMI (*n* = 337,199; Fig. 2C and Table S4). Using the functional mapping and annotation tool for GWAS analysis [33], we identified 104 genes significantly associated with FI after adjusting for BMI (Bonferroni correction, *P <* 2.8E−6; Fig. 2D and Table S5). Among the top 10 genes, only *SH2B1* (*z* = 6.05, *P* = 7.3E−10) exhibited associations with both BMI and TFM. Despite observing genomic inflation (λ_GC_ = 1.35) in the UKBB cohort analysis, we chose not to adjust for it because the linkage disequilibrium (LD) score intercept (1.05) suggested that the genomic inflation was primarily attributed to polygenicity but not population stratification [34].

**Fig. 3.**
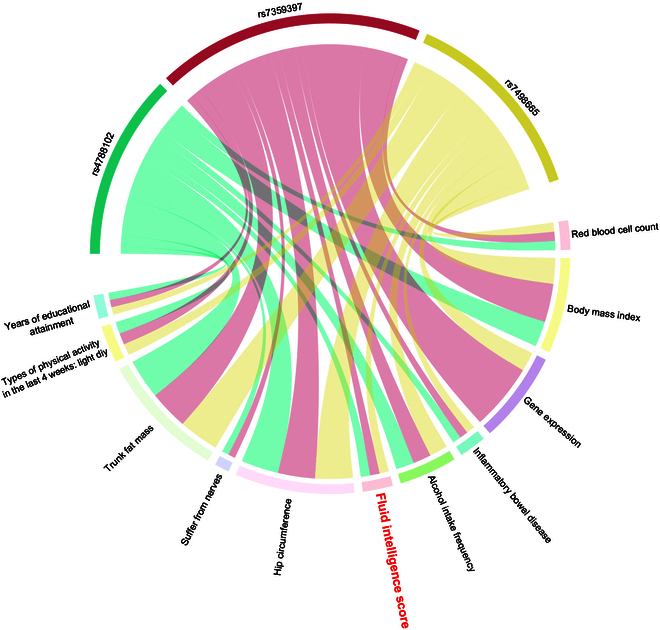
The correlations between intelligence-related *SH2B1* variants and other traits. The width of the bars for each trait/SNP (rs4788102, rs7359397, and rs7498665) of *SH2B1* indicates the relative significance in the genome-wide association.

We identified 3 SNPs in *SH2B1* (rs4788102: β = −0.06, *P* = 1.4E−9; rs7498665: β = −0.06, *P* = 1.6E−9; rs7359397: β = −0.06, *P* = 9.0E−10, Fig. S1 and Table S6) significantly associated with FI. Notably, rs7498665, a missense variant, has been linked to obesity and type 2 diabetes in population-based studies [35–37], suggesting its potential involvement in the regulation of body weight with neuronal associations [13]. The other 2 SNPs are intronic and have shown correlations with obesity measures in previous GWASs [12,13,37,38]. Data from PhenoScanner indicated that rs7498665 and rs7359397 were correlated with *SH2B1* gene expression in human tissues (Fig. 3). However, the associations of these 3 SNPs with 4 other cognitive traits, including pair matching, reaction time, prospective memory, and numeric memory, did not reach significance (Table S6).

### The correlation between *SH2B1* and FI cannot be explained solely by its associations with other traits

Despite observing associations of these 3 SNPs with traits such as alcohol intake and physical activity (Fig. 3), controlling for additional covariates, including alcohol intake frequency, hip circumference, TFM, suffering from nerves, and types of physical activity in the last 4 weeks, did not eliminate the significant genetic association between *SH2B1* polymorphisms and FI (Table S7).

Given the previously reported strong association between S*H2B1* and diabetes risk [35–37], we further controlled for diabetes diagnosis. Even after adjustment for diabetes diagnosis, the genetic associations between *SH2B1* polymorphisms and FI remained significant in the UKBB sample (Table S7). However, it is important to note that the Homeostatic Model Assessment for Insulin Resistance (HOMA-IR) could not be included in our analysis because the UKBB cohort lacked the fasting insulin and fasting glucose data, which are required for its calculation. Consequently, HOMA-IR was not included as an obesity-related covariate in our study.

It is also worth noting that educational attainment, which has a high genetic correlation with intelligence, has been used as a proxy measure in previous studies [9]. To assess the effect of educational attainment on FI within the cohort, we performed a genetic correlation analysis between *SH2B1* and FI, adjusting for educational attainment. The results showed a persistent and significant association between *SH2B1* and FI (rs4788102: β = −0.03, *P* = 5.0E−3; rs7359397: β = −0.03, *P* = 3.4E−3; rs7498665: β = −0.03, *P* = 7.1E−3; Table S8). As a result, educational attainment was not included as one of the covariates in the final analysis, indicating that the genetic association between *SH2B1* and FI is independent of educational attainment.

### Hippocampal volume serves as a mediator in the relationship between *SH2B1* and FI

**Fig. 4.**
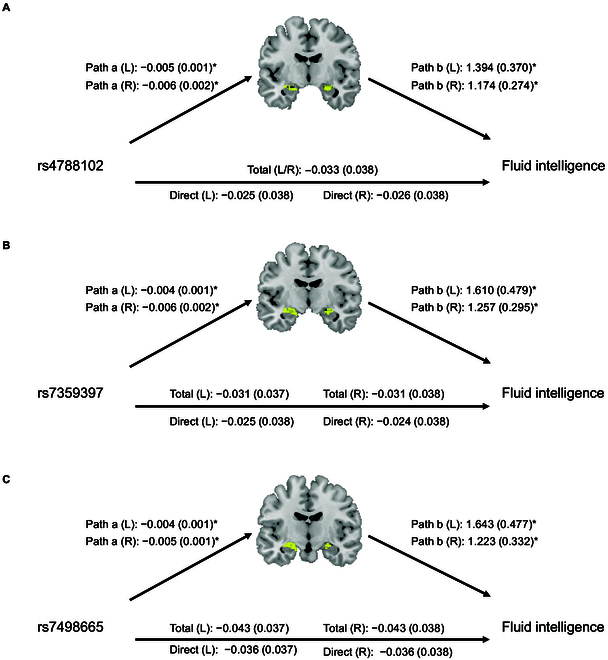
Mediation results for rs4788102, rs7359397, and rs7498665 in *SH2B1* in the bilateral hippocampi with BMI controlled for in the analysis. The path diagram shows the mediation model, with age at the imaging visit, sex, BMI at the imaging visit, the top 15 PCs, and the eTIV as a percentage of the cranial volume adjusted for. Significant regions (*P <* 0.001, 2-tailed, and 3 contiguous voxels in each of paths a, b, and a*b) mediating the correlation between rs7498665 (A), rs4788102 (B), and rs7359397 (C), respectively, and fluid intelligence scores. *P < 0.001, 2-tailed, 1,000 bootstraps.

To identify the key brain regions associated with *SH2B1* and their potential role in FI, we performed an association analysis between *SH2B1* polymorphisms and voxel-based morphometry (VBM). We observed that the hippocampus was included in the clusters that survived false discovery rate (FDR) correction with voxel-wise threshold (*q* < 0.05, Fig. S2). Given the well-established importance of the hippocampus in FI [39] and its predictive ability for FI in adult human populations [23,24], we used the Multilevel Mediation and Moderation (M3) toolbox to examine whether the association between *SH2B1* and FI was influenced by hippocampal gray matter volume at the voxel level (left: 2,184 voxels; right: 2,304 voxels) (*n* = 6,993) [40]. After AlphaSim correction, we found that the genetic association between *SH2B1* polymorphisms and FI was mediated by significant clusters in the hippocampus (left hippocampus: rs4788102: β = −0.008, *P* = 5.0E−4 at the peak voxel *x* = −20, *y* = −9, *z* = −13; rs7359397: β = −0.008, *P* = 5.0E−4 at the peak voxel *x* = −21, *y* = −9, *z* = −12; rs7498665: β = −0.010, *P* = 4.0E−4 at the peak voxel *x* = −21, *y* = −12, *z* = −13; right hippocampus: rs4788102: β = −0.009, *P* = 5.0E−4 at the peak voxel *x* = 22, *y* = −4, *z* = −16; rs7359397: β = −0.008, *P* = 5.0E−4 at the peak voxel *x* = 25, *y* = −6, *z* = −19; rs7498665: β = −0.008, *P* = 5.0E−4 at the peak voxel *x* = 25, *y* = −10, *z* = −13; Fig. S3 and Table S9), particularly in the CA1 region (Table S10), which is involved in intelligence-related information processing [41]. Specifically, our analysis revealed significant associations between the rs4788102, rs7359397, and rs7498665 variants and a reduction in hippocampal gray matter volume. Furthermore, this reduction in hippocampus volume was associated with lower FI scores. The mediation effects remained significant even after controlling for BMI in the mediation analysis (Fig. 4 and Table S11). Further mediation analysis using SPSS revealed that the mediation proportion of rs7498665 was approximately 23%, which decreased to 21% when BMI was controlled in the analysis (Table S12).

To explore the potential involvement of obesity in the *SH2B1*-mediated regulation of FI, we performed additional analyses focusing specifically on normal weight individuals (*n* = 109,457) from the UKBB cohort. The association between the 3 SNPs and FI remained significant (rs4788102: *t* = −3.36, β ± standard error of the mean [SEM] = −0.05 ± 0.02, *P* = 7.0E−4; rs7359397: *t* = −3.38, β ± SEM = −0.05 ± 0.02, *P* = 7.0E−4; rs7498665: *t* = −3.40, β ± SEM = −0.05 ± 0.02, *P* = 7.0E−4; Table S13), indicating that the correlation between the 3 SNPs in *SH2B1* and FI, mediated by the hippocampal regions, was independent of obesity.

In an exploratory analysis, we examined the association between functional connectivity of default mode and frontoparietal networks (functionally segregated neural correlates of intelligence) and *SH2B1* genotype. The analysis revealed a significant correlation between *SH2B1* genotype and default mode and frontoparietal networks (Table S14). In addition, we calculated the Euclidean distance between the MNI coordinates of the mediated cluster in the hippocampus (found in the mediation analysis) and the center of 264 brain regions within Power’s parcellation. We identified 6 clusters located in 4 hippocampus-related regions. Using Pearson’s correlation, we examined the blood-oxygen-level-dependent signal of these 4 regions of interest in relation to the other 260 brain regions, resulting in a hippocampus-related functional connectivity matrix. We found that rs4788012 was associated with the hippocampus-related sensory/somatomotor hand network (β = −0.017, *P* = 8.8E−06, FDR correction), the default mode network (β = −0.017, *P* = 3.2E−04, FDR correction), and the visual network (β = −0.018, *P* = 3.7E−04, FDR correction, Table S15). However, no significant mediation effects were found in 3 brain regions of the default mode network, including the frontal medial orbital cortex, frontal superior medial cortex, and posterior cingulate cortex (Fig. S4). In addition, no significant mediation was found in the control region (i.e., the precentral gyrus, Fig. S4). These results indicate that the hippocampus exerts a distinct role in mediating the association between *SH2B1* and FI, independent of other brain regions.

### Intelligence-related behaviors in mice are dependent on neuronal Sh2B1 in the hippocampus

**Fig. 5.**
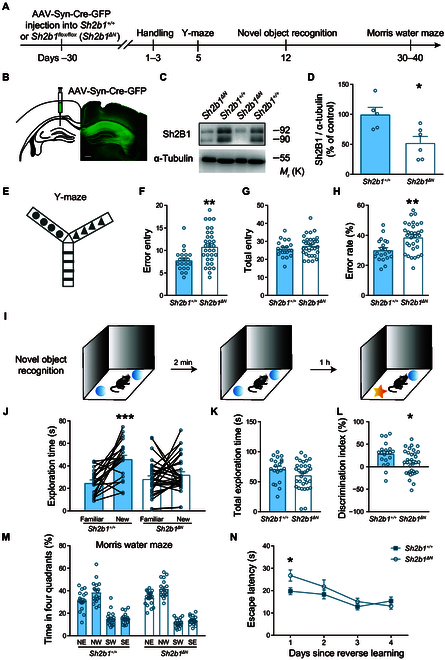
Intelligence-related behaviors in mice are dependent on neuronal *Sh2b1* expression in the hippocampus. (A) Experimental timeline. (B) Schematics of AAV injections and representative images of GFP expression in injected areas. Scale bar, 200 μm. (C and D) Representative immunoblots (C) and pooled data (D). *Sh2b1^+/+^*, *n* = 5; *Sh2b1^ΔΝ^*, *n* = 6. *P* = 0.0149 (*), unpaired Student’s *t* test. (E) Diagram of the Y-maze test. (F to H) Error entries (F), total entries (G), and error rate (%, H). *Sh2b1^+/+^*, *n* = 20; *Sh2b1^ΔΝ^*, *n* = 32. *P* = 0.0037 (**), 0.2535, and 0.015 (**), for (F), (G), and (H), *Sh2b1^+/+^* versus *Sh2b1^ΔΝ^*, unpaired Student’s *t* test. (I) Diagrams for novel object recognition test. (J to L) Novel object recognition test. *Sh2b1^+/+^*, *n* = 20; *Sh2b1^ΔΝ^*, *n* = 32. (J) Left: *Sh2b1^+/+^*, *P* = 1.0E−4 (***); Right: *Sh2b1^ΔΝ^*, *P* = 0.3014, Familiar versus New, paired Student’s *t* test. (K and L) *P* = 0.1114 and 0.0128 (*), for (K) and (L), *Sh2b1^+/+^* versus *Sh2b1^ΔΝ^*, unpaired Student’s *t* test. (M and N) Morris water maze test. (M) Percentage of time spent in the 4 quadrants during the probe trials after learning. (N) Reverse learning curve. *Sh2b1^+/+^*, *n* = 18; *Sh2b1^ΔΝ^*, *n* = 19. Two-way repeated measures ANOVA, main effect of group, *F*_1,35_ = 2.577, *P* = 0.1174. *P* = 0.0246 (*), 0.3508, 0.2751, and 0.3114, from the first to the fourth day since reverse learning, *Sh2b1^+/+^* versus *Sh2b1^ΔΝ^*, unpaired Student’s *t* test.

Building on findings from GWAS and brain imaging studies, we investigated the conservation and neurobiological basis of Sh2B1-mediated regulation of intelligence across species. To investigate the specific contribution of hippocampal neuronal *Sh2B1* to intelligence-related behaviors, we used floxed *Sh2b1* (*Sh2b1^flox/flox^*) mice to minimize the impact of peripherally distributed Sh2B1 on body metabolism [42]. To achieve region- and neuron-specific knockout of *Sh2b1*, we used adeno-associated virus (AAV) carrying both Cre recombinase and GFP under the control of the human synapsin promoter (AAV-Syn-Cre-GFP). These injections were targeted to both sides of the hippocampal CA1 region of *Sh2b1^flox/flox^* mice (*Sh2b1^ΔΝ^*). Control mice with nonfloxed *Sh2b1* alleles (*Sh2b1^+/+^*) received identical injections (Fig. 5A to D and Fig. S9A).

Our study demonstrated the essential role of hippocampal Sh2B1 in intelligence-related behaviors in mice. The first behavioral assessment was the Y-maze test to evaluate the spontaneous alternation behavior. *Sh2b1^ΔN^* mice exhibited significantly fewer spontaneous alternations but increased errors (i.e., alternate arm returns) compared to *Sh2b1^+/+^* mice (Fig. 5E to H), indicating impaired working memory in the *Sh2b1^ΔN^* mice.

We then performed the novel object recognition test to assess short-term memory in the mice. The results showed that *Sh2b1^ΔN^* mice exhibited impaired short-term object recognition memory compared to *Sh2b1^+/+^* mice, as indicated by their lower object discrimination indices (Fig. 5I to L). Importantly, this difference could not be attributed to variations in exploratory drive, as the total object interaction time was comparable between the 2 groups (Fig. 5K).

To investigate the specific role of *Sh2B1* in intelligence rather than general learning ability, we used the Morris water maze test. The results showed that both *Sh2b1^ΔN^* and *Sh2b1^+/+^* mice exhibited similar latencies to locate the platform during the learning sessions and showed comparable preferences for the target quadrant during the probe test (Fig. S5 and Fig. 5M), suggesting that hippocampal loss of Sh2B1 did not affect hippocampus-dependent spatial learning and memory. However, in the reversal learning ability test, *Sh2b1^ΔN^* mice took longer to find the platform (Fig. 5N), indicating impaired behavioral flexibility when the platform’s location was changed.

Finally, we subjected *Sh2b1^+/+^* and *Sh2b1^ΔN^* mice to behavioral paradigms assessing basal locomotor activity and anxiety, including the open field and elevated zero maze tests. The results showed no significant differences between the 2 groups in the distance traveled in the open field (Fig. S6A), and deletion of *Sh2b1* in the hippocampus did not induce anxiogenic or anxiolytic effects (Figs. S6B to D and S7).

In addition, we used another cohort of *Sh2b1^ΔN^* mice and employed *Sh2b1^flox/ flox^* mice injected with AAV-Syn-GFP as controls (*Sh2b1^N-Ctrl^*), which yielded similar results (Fig. S8). Taken together, our results suggest that hippocampal Sh2B1 plays a specific role in working memory, short-term novel object recognition memory, and behavioral flexibility, while leaving general learning abilities, basal locomotor activity, and anxiety unaffected.

### Loss of Sh2b1 in hippocampal inhibitory neurons, but not in excitatory neurons, impairs intelligence

**Fig. 6.**
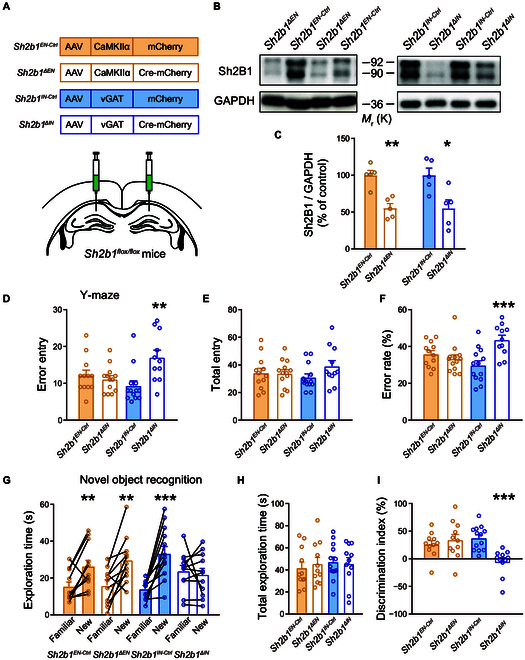
Mice null for *Sh2b1* in inhibitory neurons, but not excitatory neurons, in the hippocampus exhibit impaired intelligence-related behaviors. (A) Schematics of AAV injections. (B and C) Representative immunoblots (B) and pooled data (C). *n* = 5 each group. Left: *P* = 1.1E−3 (**), *Sh2b1^ΔΕΝ^* versus *Sh2b1^ΕΝ-Ctrl^*; Right: *P* = 0.0175 (*), *Sh2b1^ΔIΝ^* versus *Sh2b1^IΝ-Ctrl^*, unpaired Student’s *t* test. (D to F) Y-maze test. *Sh2b1^ΕΝ-Ctrl^*, *n* = 12; *Sh2b1^ΔEΝ^*, *n* = 13. *Sh2b1^IΝ-Ctrl^*, *n* = 13; *Sh2b1^ΔIΝ^*, *n* = 11. *P* = 0.5307, 0.9571, and 0.4031, for error entries (D), total entries (E), and error rate (%, F), respectively, *Sh2b1^ΔEΝ^* versus *Sh2b1^ΕΝ-Ctrl^*, unpaired Student’s *t* test. *P* = 3.7E−3 (**), 0.1052, and 9.0E−4 (***), for error entries (D), total entries (E), and error rate (%, F), respectively, *Sh2b1^ΔIΝ^* versus *Sh2b1^IΝ-Ctrl^*, unpaired Student’s *t* test. (G to I) Novel object recognition test. *Sh2b1^ΕΝ-Ctrl^*, *n* = 11; *Sh2b1^ΔEΝ^*, *n* = 11. *Sh2b1^IΝ-Ctrl^*, *n* = 13; *Sh2b1^ΔIΝ^*, *n* = 11. (G) Exploration time for each object. *P* = 6.5E−3 (**), 3.4E−3 (**), 2.0E−4 (***), and 0.6273, Familiar versus New, for the *Sh2b1^ΕΝ-Ctrl^*, *Sh2b1^ΔΕΝ^*, *Sh2b1^IΝ-Ctrl^*, and *Sh2b1^ΔIN^* mice, respectively, paired Student’s *t* test. (H) Total exploration time. *P* = 0.6403 and 0.8206, for *Sh2b1^ΕΝ-Ctrl^* versus *Sh2b1^ΔΕΝ^* and *Sh2b1^IΝ-Ctrl^* versus *Sh2b1^ΔIN^*, respectively, unpaired Student’s *t* test. (I) Discrimination index. *P* = 0.5120 and 1.0E−4 (***), for *Sh2b1^ΕΝ-Ctrl^* versus *Sh2b1^ΔΕΝ^* and *Sh2b1^IΝ-Ctrl^* versus *Sh2b1^ΔIN^*, respectively, unpaired Student’s *t* test.

In order to identify the specific cell type responsible, we used an AAV vector to simultaneously express Cre recombinase and mCherry under the control of the Ca^2+^/calmodulin-dependent protein kinase type II subunit α or the vesicular γ-aminobutyric acid (GABA) transporter (VGAT) promoter in *Sh2b1^flox/flox^* mice (Fig. S9B and C). This allowed us to achieve region- and cell-type-specific knockout of *Sh2b1* in excitatory (*Sh2b1^ΔEN^*) or inhibitory (*Sh2b1^ΔIN^*) neurons (Fig. 6A to C and Fig. S10). The *Sh2b1^ΔIN^* mice exhibited higher error rates in the Y-maze test (Fig. 6D to F) and failed to show a significant preference in the novel object recognition test (Fig. 6G to I) compared to the control group (*Sh2b1^IN-Ctrl^*). These results suggest that loss of *Sh2b1* specifically in GABAergic neurons severely impairs working memory and short-term memory, respectively. However, no differences were observed between the *Sh2b1^ΔEN^* and its control group, *Sh2b1^EN-Ctrl^* mice (Fig. 6D to I). Furthermore, we analyzed the potential correlation between the behavioral phenotype and the expression level of Sh2B1 in different groups (Fig. 6 and Fig. S8), revealing a significant correlation between intelligence performance and the expression level of Sh2B1 in mice with pan-neuronal manipulation of Sh2B1, with a tendency for association in inhibitory-neuron-specific but not excitatory-neuron-specific manipulation (Fig. S11). These results suggest that Sh2B1 plays a crucial role in intelligence regulation specifically within inhibitory neurons in the hippocampus, while its role in excitatory neurons does not appear to have a marked impact on intelligence.

Furthermore, we investigated whether *Sh2b1* deletion indirectly affects metabolism. We evaluated several metabolic parameters in *Sh2b1^ΔIN^* mice but found no significant differences compared to the control group, including oxygen consumption (VO_2_), carbon dioxide production (VCO_2_), respiratory exchange ratio, physical activity, and energy expenditure (Fig. S12). Moreover, we observed no significant difference in body weight between *Sh2b1^ΔIN^* and *Sh2b1^IN-Ctrl^* mice (22.92 ± 0.43 g and 23.03 ± 0.41 g, respectively, mean ± SEM, *P* = 0.8535, unpaired Student’s *t* test, *n* = 8 each group, *Sh2b1^ΔIN^* versus *Sh2b1^IN-Ctrl^* mice). Additionally, we conducted glucose tolerance test (GTT) in addition to insulin tolerance test (ITT) to assess metabolic function. The results showed that *Sh2b1* deletion in inhibitory neurons did not affect glucose metabolism in mice (Fig. S13), implicating that the effects of Sh2B1 on intelligence-related behaviors are largely independent of metabolism.

To investigate the focal differences in brain anatomy in *Sh2b1^ΔIN^* compared to *Sh2b1^IN-Ctrl^* mice, we performed VBM using magnetic resonance imaging (MRI). Comprehensive morphometric assessment of the whole brain did not reveal any significant difference in the volume of the hippocampus and other brain regions between the 2 groups (Fig. S14). This inconsistency with human data may be due to the acute manipulation of Sh2B1 expression in adult mice, in contrast to the natural *SH2B1* polymorphisms and associated hippocampal volumes observed in in humans.

**Fig. 7.**
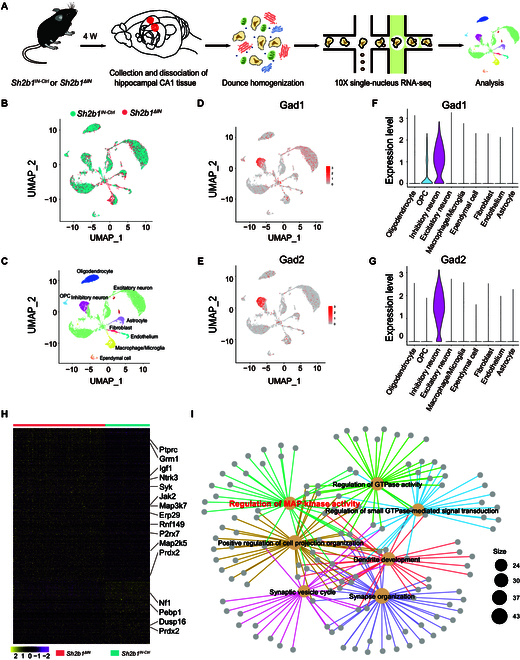
Single-nucleus gene expression analysis of *Sh2b1* conditional depletion in mouse hippocampal inhibitory neurons. (A) Experimental design. (B and C) UMAP visualization of 13,603 cells colored by cluster (9 major clusters, names indicated), sampled. Dots, individual cells; colors, neuron clusters. (D to G) Feature violin plot and UMAP plot show the cell-type-specific marker genes *Gad1* (D and F) and *Gad2* (E and G) in the inhibitory neurons. (H) Heatmap of genes with differential expression between pairwise comparison of 330 *Sh2b1^IN-Ctrl^* and 370 *Sh2b1^ΔIN^* inhibitory neuron nuclei. (I) Dot plot graph represents top GO terms enriched for differentially expressed genes in pairwise comparison of *Sh2b1^ΔIN^* versus *Sh2b1^IN-Ctrl^* inhibitory neuron nuclei for 10x Genomics.

To gain insight into the effects of hippocampal Sh2B1 across mouse cell types, we conducted single-nucleus RNA sequencing (snRNAseq) on the hippocampi of *Sh2b1^ΔIN^* and *Sh2b1^IN-Ctrl^* mice (Fig. 7A). By profiling 13,603 individual nuclei and identifying 23 distinct clusters (Fig. 7B to G and Figs. S15 to S18), we observed that differentially expressed genes between inhibitory neuronal clusters in the *Sh2b1^IN-Ctrl^* and *Sh2b1^ΔIN^* mice were significantly enriched in functional classes associated with the regulation of MAP kinase activity, synaptic vesicle cycle, and synapse organization, among others (Fig. 7H and I and Fig. S19). These results are consistent with the known role of Sh2B1 as an adaptor protein that influences various signaling pathways [30].

### Sh2B1 modulates hippocampal ERK signaling to modulate intelligence

**Fig. 8.**
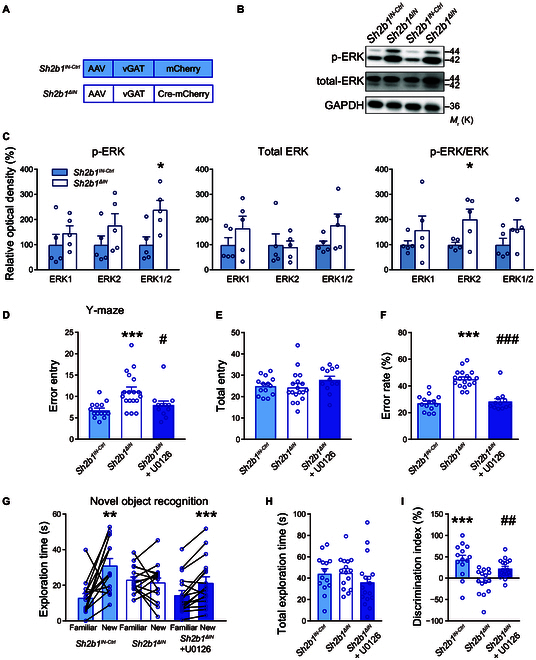
*Sh2b1* regulates intelligence through hippocampal ERK signaling. (A) Schematics of AAV injections. (B and C) Representative immunoblots (B) and pooled data (C). *n* = 5 each group. Left: p-ERK, *P* = 0.3813, 0.2518, and 0.0195 (*); Middle: total ERK, *P* = 0.2687, 0.8625, and 0.1297; Right: the ratio of p-ERK and total ERK, *P* = 0.3349, 0.0372 (*), and 0.1683, *Sh2b1^IΝ-Ctrl^* versus *Sh2b1^ΔIΝ^*, for ERK1, ERK2, and ERK1/2, respectively, unpaired Student’s *t* test. (D to I) Effects of pharmacological inhibition of ERK on the behavioral performance. (D to F) Y-maze test. *Sh2b1^IN-Ctrl^*, *n* = 14; *Sh2b1^ΔIΝ^*, *n* = 18; *Sh2b1^ΔIΝ^* + U0126, *n* = 12. (D) *P* = 9.0E−4 (***), *Sh2b1^IN-Ctrl^* versus *Sh2b1^ΔIΝ^*; ^#^*P* = 0.0324, *Sh2b1^ΔIΝ^* versus *Sh2b1^ΔIΝ^* + U0126; (E) *P* = 0.8006, *Sh2b1^IN-Ctrl^* versus *Sh2b1^ΔIΝ^*; *P* = 0.1840, *Sh2b1^ΔIΝ^* versus *Sh2b1^ΔIΝ^* + U0126; (F) *P* = 4.2E−9 (***), *Sh2b1^IN-Ctrl^* versus *Sh2b1^ΔIΝ^*; *P* = 3.6E−7 (^###^), *Sh2b1^ΔIΝ^* versus *Sh2b1^ΔIΝ^*+ U0126, unpaired Student’s *t* test. (G to I) Novel object recognition test. *Sh2b1^IN-Ctrl^*, *n* = 15; *Sh2b1^ΔIΝ^*, *n* = 18; *Sh2b1^ΔIΝ^* + U0126, *n* = 17. (G) *P* = 3.4E−3 (**), 0.5709, and 7.0E−4 (***), Familiar versus New, for the *Sh2b1^IΝ-Ctrl^*, *Sh2b1^ΔIN^*, and *Sh2b1^ΔIΝ^* + U0126 mice, respectively, paired Student’s *t* test. (H) *P* = 0.8917 and 0.2118, for *Sh2b1^IΝ-Ctrl^* versus *Sh2b1^ΔIN^* and *Sh2b1^ΔIΝ^* versus *Sh2b1^ΔIΝ^* + U0126, respectively, unpaired Student’s *t* test. (I) *P* = 4.0E−4 (***) and 2.4E−3 (^##^), for *Sh2b1^IΝ-Ctrl^* versus *Sh2b1^ΔIN^* and *Sh2b1^ΔIΝ^* versus *Sh2b1^ΔIΝ^* + U0126, respectively, unpaired Student’s *t* test.

Building on these findings, we sought to examine the downstream signaling pathways of hippocampal Sh2B1 that regulate intelligence-related behaviors, with a particular focus on extracellular signal-regulated kinases (ERKs), which are the final effectors of the MAPK cascade. We found that mice with either hippocampal neuronal loss of *Sh2b1* (Fig. S20) or inhibitory neuronal loss of *Sh2b1* (Fig. 8A to C) exhibited significantly increased phosphorylation levels of ERK1 and ERK2 compared to their respective control groups. In addition, we examined the phosphorylation changes of other classical downstream signaling pathways, p38 MAPK, c-Jun N-terminal kinase, and the upstream kinase of ERK, MAP kinase (MAPKK or MEK), upon *Sh2b1* deletion. No significant differences were observed in these pathways (Fig. S21). These results suggest that there is a specific and selective association between Sh2B1 and the ERK signaling pathway.

To investigate whether reducing ERK activity could reverse the intelligence-related impairment resulting from genetic loss of *Sh2b1*, we selectively treated *Sh2b1^ΔIN^* mice with the ERK inhibitor U0126 in the hippocampus. Remarkably, this intervention resulted in a significant improvement in the performance of mice in working memory tasks (Fig. 8D to F) as well as short-term memory tasks (Fig. 8G to I). Therefore, these collective results suggest that Sh2B1 plays a crucial role in intelligence-related behaviors in both humans and mice, primarily exerting its effects in the hippocampus and may exhibit cell-type specificity in the regulation of MAPK signaling cascades.

## Discussion

Obesity is a multifactorial and chronic metabolic disorder that negatively affects cognitive performance, as evidenced in human-based studies [43]. Recent studies [31,32] have shown that *SH2B1*, a gene implicated in leptin and insulin signaling [44], is a substantial contributor to the genetic architecture of obesity. *SH2B1* has been identified as a susceptibility locus for obesity in several BMI- or obesity-related GWASs in different human cohorts [12,13,16]. Here, we further investigated *SH2B1* as a genetic locus underlying the association between obesity and FI. First, we confirmed in the UKBB database that *SH2B1* is both an obesity susceptibility locus and a gene with an overlapping effect on FI in the same cohort, using records for GWAS on BMI and TFM, 2 anthropometric traits associated with obesity. Second, our analysis showed that *SH2B1* remained on the list of genes associated with FI after controlling for BMI, suggesting that *SH2B1* may be an independent genetic factor associated with FI that can influence both metabolism and FI. Third, we found that SNPs in *SH2B1* were not associated with other cognitive traits, suggesting that Sh2B1 plays a specific rather than a general role in intelligence. Fourth, our analysis showed that SNPs in *SH2B1* were associated with BMI, hip circumference, and TFM, which are direct indicators of obesity, as well as alcohol intake [45] and physical activity, which may indirectly contribute to obesity, suggesting that these lifestyle-related traits may also be linked to common genetic mechanisms. However, by controlling for these additional obesity-related covariates for intelligence in the GWAS using the UKBB cohort, we found that the genetic association between the identified *SH2B1* polymorphisms and FI remained significant, strengthening the independence of *SH2B1* as a genetic factor associated with FI. Fifth, our analysis showed that the association between *SH2B1* and intelligence remained significant even when normal weight individuals were analyzed, suggesting that obesity is not a prerequisite for the association between *SH2B1* and FI. Sixth, our genetic-behavioral correlation analysis with MRI data revealed that the effect of *SH2B1* genetic variation on FI was partially mediated by its association with bilateral hippocampal volume, which is consistent with hippocampal volume being a predictor of humoral intelligence in adult human populations, including the elderly [23,24]. In summary, *SH2B1* genetic variation in human populations, has independent, negative consequences for FI that are partially mediated by the effect of the variants on hippocampal structure, establishing previously unknown genetic and neural correlates linking metabolism and intelligence.

The identification of Sh2B1 [46,47] and its associated signaling cascades, along with their respective cell-type-specific physiological importance may provide a mechanistic framework for understanding its newly identified role in intelligence. Sh2B1 family proteins are known to encompass multiple modular protein–protein interaction domains, which enable them to serve as connectors between various regulatory and effector proteins [30]. The distinct specificities of these domains allow the Sh2B1 protein to elicit diverse individual responses to specific signals [30]. Sh2B1 has been implicated in various signaling pathways mediated by receptor tyrosine kinases, including the insulin receptor [47] and IGF-I [48], as well as the JAK family of non-receptor tyrosine kinases [49] in response to ligand binding to growth hormone [46] and leptin receptors. These Sh2B1-mediated signaling cascades likely underlie its role in modulating the body’s sensitivity to leptin or insulin to regulate energy production, glucose homeostasis, and body weight. Supporting this, *Sh2b1-*deficient animals develop obesity and metabolic syndrome [50–53], while humans with *SH2B1* loss-of-function mutations present with hyperphagia, childhood-onset obesity, disproportionate insulin resistance, and reduced adult height [54]. In terms of metabolic control in specific tissues and cell types, Sh2B1’s expression in both central nervous system neurons *[*44,55] and peripheral non-neuronal cells [42,52,56] is crucial for protection against obesity and metabolic diseases.

In contrast to Sh2B1’s role in metabolism, its involvement in intelligence is predominantly attributed to its function in the central nervous system. We revealed that Sh2B1 expression in inhibitory neurons, specifically in the hippocampal CA1 region, plays a pivotal role in intelligence-related behaviors in mice. Notably, the effects of hippocampal interneuron Sh2B1 on intelligence-related behaviors are largely independent of body metabolism. Mechanistically, we observed a significant enhancement in ERK activity upon *Sh2b1* gene deletion, while the activity of other classical downstream signaling pathways, such as p38 MAPK or c-Jun N-terminal kinase, remained unaffected. The ERK MAP kinase signaling cascade is known to play a fundamental role in brain development, learning, and memory [57,58], and the altered ERK activity contributes to the Sh2B1-mediated regulation of intelligence behaviors. Supporting this, pharmacological inhibition of ERK signaling reversed the behavioral deficits caused by *Sh2b1* knockout, highlighting the crucial involvement of altered ERK activity in Sh2B1-dependent regulation of intelligence behaviors. Interestingly, these mechanisms parallel findings in a mouse model of the learning disabilities associated with neurofibromatosis type I (NF1), where *Nf1* heterozygous null mutants exhibit enhanced ERK signaling and increased GABA release in the hippocampus. Moreover, pharmacological downregulation of ERK signaling and subthreshold doses of GABA(A) receptor antagonists can effectively reverse this defect [59].

Sh2B1 is known to be involved in neurotrophic factor-related signaling pathways [60–63], including those associated with nerve growth factor (NGF), BDNF, and glia cell line-derived neurotrophic factor, which may contribute to its regulatory effect on ERK signaling and intelligence-related behaviors. Upon NGF stimulation, Sh2B1 undergoes phosphorylation at multiple sites by kinases within the ERK cascade [64]. However, the precise mechanisms by which Sh2B1 transduces the reverse signal to regulate ERK signaling remain unexplored. In our study, we made a discovery that Sh2B1 exerts a negative regulatory influence on ERK activity in hippocampal neurons, providing novel insights into the molecular mechanisms underlying Sh2B1’s role in the regulation of intellectual functions.

To elucidate the molecular basis of Sh2B1’s regulation of ERK activity, we examined the phosphorylation changes of MAPKK or MEK, an upstream kinase of ERK, upon *Sh2b1* deletion. Surprisingly, we did not observe significant differences, suggesting that there might be a more specific or direct interaction between Sh2B1 and ERK. However, further investigations are needed to fully understand the molecular basis of this potential interaction. In summary, we propose a working model for the Sh2B1-mediated regulation of intelligence-related behaviors, where Sh2B1 is activated in a coordinated manner, likely through the integration of multiple signaling pathways, including receptors for neurotrophic factors such as BDNF. Subsequently, Sh2B1 modulates ERK signaling and activity-dependent GABA release, leading to well-organized neuronal activity in the hippocampus. This orchestrated neuronal activity is crucial for working memory, short-term recognition memory, and behavioral flexibility, all of which are essential domains of intelligence. Nonetheless, additional research is required to further refine and deepen our understanding of the intricate molecular mechanisms underlying Sh2B1's role in the regulation of intelligence-related behaviors.

Extrapolating results from FI tests in humans to experimental studies in mice presents an ongoing challenge. However, due to the strong correlation between FI and working memory in humans [65,66], we employed the Y-maze test in mice as a means to evaluate spontaneous alternation behavior, a measure believed to reflect working memory performance. Remarkably, mice with hippocampal deletion of *Sh2b1* displayed significant behavioral deficits in the Y-maze test. While the frontal cortex is widely recognized for its involvement in working memory, growing evidence indicates the hippocampal involvement in this cognitive process as well [67–69]. Furthermore, our human data identified the influence of *SH2B1* polymorphisms not only on the hippocampal structure but also on its connection to the functional connectivity matrix. These intriguing findings led us to speculate that the hippocampus, as a subcortical node, plays a critical role in working memory, a cognitive process closely associated with FI in humans. The involvement of Sh2B1 in the hippocampus may be a key mechanism linking its genetic loss of function to working memory deficits observed in mice, suggesting the potential relevance of these findings to human intelligence. Nonetheless, further investigation is warranted to fully elucidate the precise molecular and circuitry mechanisms by which Sh2B1 affects working memory and its impact on overall intelligence.

Likewise, we observed compromised short-term memory, another distinct cognitive construct associated with FI [65], in mice with hippocampal deletion of *Sh2b1*. In contrast, the Morris water maze test, assessing hippocampus-dependent spatial learning and memory, showed only subtle differences following hippocampal deletion of *Sh2b1*, primarily affecting behavioral flexibility rather than general learning abilities. These seemingly paradoxical observations might reflect a genuine cognitive function in mice that parallels the description of FI in humans, encompassing abstract reasoning, problem solving, and adaptation to a dynamic environment. Nevertheless, the identification of hippocampal Sh2B1 in intelligence-related behaviors in mice does not negate the potential role of Sh2B1 in other brain regions, warranting further comprehensive investigation in this context.

In conclusion, our study offers compelling evidence supporting the critical role of hippocampal Sh2B1 in intelligence-related behaviors, which has important implications for humans as well. The intriguing involvement of this gene in both intelligence and metabolism underscores the intricate interplay between neural and metabolic processes in brain function. Moreover, these findings hold promise for potential clinical applications in the treatment of metabolic disorders associated with intellectual disabilities. Considering the observed impact of Sh2B1 on cognitive function, future studies could explore the potential therapeutic strategies targeting Sh2B1 or its downstream pathways for the management of such conditions. In essence, our work represents a remarkable advancement in our understanding of the genetic and neural basis of intelligence, opening up new avenues for in-depth exploration of the underlying mechanisms of this fascinating and multifaceted trait.

## Materials and Methods

### Human subjects

The UKBB is a substantial and ongoing prospective cohort that involves over half a million individuals aged between 37 and 73 years, who were recruited during the period between 2006 and 2010 (http://www.ukbiobank.ac.uk/) [70]. As part of this initiative, cognitive tests and brain imaging data were meticulously collected on the same assessment day. The study was conducted with the approval of the UKBB Research Ethics Committee (REC reference 11/NW/0382) and was carried out under UKBB application 19542.

### Mice

The *Sh2b1*-floxed mice were generated as previously described [42]. Male nonfloxed (*Sh2b1^+/+^*) and homozygous floxed (*Sh2b1^flox/flox^*) littermates were obtained from *Sh2b1^flox/+^* intercrosses. At the time of the experiments, the animals were aged between 8 and 12 weeks and had no prior exposure to the experimental conditions. The mice were housed in groups of less than 5 per cage before the start of the experimental sessions. They were maintained under a 12-h light/12-h dark cycle, with lights on at 0700, and provided with food and water available ad libitum. The experiments were conducted between 0900 and 1800. All necessary efforts were taken to minimize animal discomfort and reduce the number of animals used. The Animal Ethics Committee of East China Normal University, Shanghai, China, approved all experimental protocols.

### Studies in humans

#### Fluid intelligence

FI, which is highly associated with overall intelligence [1], has been commonly employed to evaluate various levels of cognitive abilities. In our study, we used an FI test to assess participants’ reasoning and problem-solving abilities [66]. The FI test comprised a 13-item verbal–numerical reasoning assessment conducted at 2 time points: baseline (FI_1) and the first FI imaging visit (FI_MRI). Both FI_1 and FI_MRI were normally distributed and treated as quantitative variables in our analyses. FI_1 was utilized in the GWAS of FI, while FI_MRI was employed in the mediation analysis to investigate the relationships between the target SNPs, hippocampal gray matter volume, and FI scores.

#### Other cognitive traits

In addition to investigating the unique association between *SH2B1* and FI, our study considered data for 4 other cognitive tests available in the UKBB [71]. These cognitive tests included the numeric memory test, where participants recalled increasingly longer series of digits (maximum: 12) to assess memory performance (*n* = 35,661). Another test evaluated reaction time, in which participants played 12 rounds of the timed card game called “Snap”, and the mean log-transformed reaction time across trials was used for analysis (*n* = 335,139). Prospective memory was assessed by asking participants to perform a preplanned instruction after a specific interval. In this test, participants were initially asked to touch a blue square out of 4 colored symbols, whereas the correct behavior was to touch an orange circle instead. The score was binary (zero or one) based on whether participants failed on the first trial (*n* = 106,816).

Furthermore, a pairs matching test was conducted, where participants were presented with 6 pairs of cards to match as many pairs as possible in as fewest trials. The total number of errors was used as the final measure, and the data were log-transformed as log10(*X* + 1) due to the skewed distribution and zero inflation before analysis (*n* = 337,199).

#### Genetic data

Genetic data were obtained from the UKBB cohort, consisting of over 500,000 participants. Affymetrix conducted genotyping using the BiLEVE Axiom Array for approximately 50,000 participants and the Affymetrix UKBB Axiom Array was used for the remaining ~450,000 participants. To ensure data quality, variants with a minor allele frequency <0.001, a call rate <95%, and a Hardy–Weinberg equilibrium test *P* value < 1.0 × 10^−10^ were removed from the analysis.

Sample quality control was also conducted, utilizing information provided by the UKBB, including variables such as in.white.British.ancestry.subset and excess.relatives from the file ukb_sqc_v2.txt [72]. After implementing these quality control measures, a total of 337,199 samples with measured SNPs and estimated recent British ancestry were included in our study. Further details on the genetic data used in our analysis can be accessed at the UKBB’s official website (http://www.ukbiobank.ac.uk). The rigorous quality control measures undertaken ensure the reliability and integrity of the genetic data, allowing us to confidently investigate the association between *SH2B1* and intelligence-related behaviors.

#### Association analysis

To identify shared genetic loci associated with intelligence and metabolism, we accessed summary GWAS data for FI, BMI, and TFM from the PhenoScanner database (http://www.phenoscanner.medschl.cam.ac.uk). We focused on SNPs with genome-wide significance (*P <* 5.0E−10, LD: *r*^2^ ≥ 0.8) and mapped them to corresponding genes using the MAGMA software (https://ctg.cncr.nl/software/magma).

In addition, we conducted a GWAS of intelligence using linear regression modeling to investigate potential gene associations with intelligence that were independent of metabolic disorders, such as obesity. The covariates included in the modeling were gender, baseline age, baseline BMI, and 15 principal coordinates (PCs) using PLINK 1.9 (http://www.cog-genomics.org/plink/1.9/). Educational attainment, which is highly genetically correlated with intelligence and has been used as a proxy measure in previous reports [9], was not used as a covariate in this analysis. Genome-wide significance was set at *P <* 5.0E−8. To visualize the GWAS results, Manhattan and QQ plots were generated using the CMplot tool (https://github.com/YinLiLin/R-CMplot).

To assess the genomic inflation and correct for multiple testing, we used LD score regression to estimate the LD score intercept [73]. To perform genome-wide gene-based association analysis, we utilized the MAGMA software [74] and applied a Bonferroni correction for 17,825 autosomal protein-coding genes from NCBI build 37. Genes were considered to have genome-wide significance if their *P* value was less than *P* = 2.86E−6.

To further investigate the genetic loci within *SH2B1*, we calculated and plotted pairwise LD between SNPs within the gene using the R package LDheatmap. By employing these comprehensive analyses and stringent statistical criteria, we aimed to identify relevant genetic loci and elucidate the potential overlapping molecular basis of intelligence and metabolism.

#### Genetic association with other phenotypes

We utilized summary GWAS data from PhenoScanner to investigate whether SNPs within *SH2B1* gene were associated with traits or diseases beyond metabolic indices and intelligence. Specifically, we focused on SNPs with genome-wide significance (*P <* 5.0E−10) that were in high LD (*r*^2^ ≥ 0.8) with the target SNPs in *SH2B1*. By extracting association results from PhenoScanner, we aimed to identify the potential genetic effects of *SH2B1* on other phenotypes.

### Genetic association with other cognitive tests

To explore the association between *SH2B1* genotypes and cognitive performance in additional cognitive tests, including numeric memory, reaction time, pairs matching, and prospective memory, we employed 4 linear models and 1 logistic model. In these models, age, sex, and 15 PCs were included as covariates to control for potential confounding factors. By incorporating these additional cognitive tests into our analysis, we sought to further elucidate the broader impact of *SH2B1* genetic variations on cognitive function beyond FI.

#### Structural imaging and preprocessing in UKBB

Structural brain MRI data in the UKBB were acquired from a dedicated Siemens Skyra 3T scanner running VD13A SP4, equipped with a standard Siemens 32-channel RF receive head coil. The T1-weighted structural imaging protocol used a resolution of 1 × 1 × 1 mm and a field of view of 208 × 256 × 256 matrix. The duration of the scan was 5 min, employing a 3D MPRAGE sequence in the sagittal plane with in-plane acceleration iPAT = 2 and iPAT = 2 and prescan-normalization. For more detailed information about the imaging protocol, please refer to https://biobank.ctsu.ox.ac.uk/crystal/ukb/docs/brain_mri.pdf.

We performed processing on the T1 images using VBM with the VBM8 toolbox, which is based on the SPM12 package (http://www.fil.ion.ucl.ac.uk/spm). In total, T1 images from 9,888 subjects were processed, and voxel-based gray matter volumes were extracted.

To further investigate the significant findings observed in the hippocampus, we utilized the CoBrALab atlas, which subdivides the hippocampus into 7 regions: fimbria, CA1, CA2/CA3, CA4/dentate gyrus, subiculum, stratum, and alveus (https://github.com/CoBrALab/atlases/tree/master/hippocampus-subfields). As different T1 templates were used in CoBrALab (ICBM152 2009c) and the automated anatomical labeling (AAL) (Colin 27) atlases, we first spatially normalized the gray matter segment of the CoBrALab atlas to the Colin 27 standard space. We then applied the 2009c to Colin27 deformation to the CoBrALab atlas to obtain the hippocampal subregions aligned with the Colin27 T1 template. The final hippocampal subregions were resampled into 1.5 mm^3^ cubic voxels to extract regional gray matter volume information for further analysis in the study group.

#### Mediation analysis

Mediation analysis was conducted using the Mediation Toolbox (https://github.com/canlab/MediationToolbox) with a 1,000 bias-corrected bootstrap sample for significance testing. The bilateral hippocampal voxel-based volume was considered as the proposed mediator, and its mediation effect of the association between the intelligence-associated SNPs within *SH2B1* and FI was assessed. The mediation analyses were adjusted for the effects of age at imaging, sex, estimated total intracranial volume (eTIV), and the 15 PCs. Additionally, BMI at the time of imaging was controlled to determine whether the mediation effect was independent of obesity-related metabolic abnormalities.

To identify significant clusters with significant mediation effects, we applied Monte Carlo simulation-based AlphaSim correction. The threshold for individuals was set at *P <* 0.005, and the cluster level significance was set at *P <* 0.05, with a minimum contiguous voxel size of 3 and the smoothness of the data set to 9 mm. This correction was used to account for multiple comparisons between the hippocampal voxels.

In cases where the identified clusters showed some overlapping voxels, we calculated the total volume of these overlapping voxels and reanalyzed the mediation using SPSS version 24.0 (SPSS, Inc., Chicago, IL, USA). This allowed us to quantify the extent to which the *SH2B1*–intelligence association was mediated. The analysis was adjusted for age at imaging, sex, eTIV, and the 15 PCs as covariates. BMI at the time of imaging was also included as a covariate to consider potential metabolic effects.

For resting-state functional magnetic resonance imaging, identical acquisition parameters were used, with a spatial resolution of 2.4 mm and a TR of 0.735 s with an acceleration factor of 8 using a multiband accelerator. To explore possible mediation effects in additional cortical regions, especially within the default mode network, voxel-wise mediation analyses were performed for the frontal superior medial cortex, the frontal medial orbital cortex, and the posterior cingulate cortex. As a control region for the mediation analysis, the precentral gyrus, which has not been previously implicated in intelligence, was also included.

### Studies in mice

#### Surgery and virus microinjection in mice

Mice aged 4 to 5 week were anesthetized using 1% sodium pentobarbital via a single intraperitoneal injection (10 ml/kg of body weight) and secured in a stereotaxic frame (RWD Life Science, China) for virus injection. Viral vector preparations with titers exceeding 1 × 10^12^ viral genome-containing particles/ml were bilaterally administered into the hippocampus. The stereotaxic coordinates for injection were determined based on the mouse brain atlas [75] with anteroposterior at −2.20 mm, lateral at ±2.00 mm, and dorsoventral at −1.60 mm. Four weeks after injection procedure, behavioral experiments were conducted.

Microelectrodes connected to a microinjector pump (KDS 310, KD Scientific, USA) were used to deliver a volume of 0.7 μl to each side of hippocampal CA1 at a rate of 0.1 μl/min during injection. Following the experiment, the injection sites were examined to ensure the precise placement of the cannulas. Mice with cannulas that were not positioned correctly excluded from the data analysis. In order to confirm the successful delivery of the viral vector, brain slices from animals treated with the virus were examined under fluorescence microscopy.

#### Guide cannula implantation and drug injection

The mice were continuously anesthetized with 1% sodium pentobarbital (10 ml/kg body weight) during the implantation of guide cannula and drug injection procedures. Using the mouse brain atlas [75], a bilateral guide cannula (Plastics One, USA) measuring 26 gauge was placed 1 mm above the target region dorsal to the hippocampal CA1. The coordinates of the target region were 2.2 mm posterior to bregma, 2 mm mediolateral, and 1 mm ventral to the cranial surface. To avoid clogging, a stylus was inserted into the guide cannula during the implantation process. After the implantation process, the mice were given a recovery period of 1 week before any experimental manipulation.

Microinfusions were performed by replacing the stylus with a 30-gauge infusion cannula that extended 1 mm from the tip of the guide cannula to target hippocampal CA1. A microsyringe was connected to the infusion cannula through PE20 tubing and was then driven by a KDS 310 microinfusion pump. Mice that had cannulas erroneously placed were excluded from the data analysis. One hour prior to behavioral testing, mice were injected in situ with either U0126 (Promega, USA, catalog no. V1121) dissolved in 10% DMSO or vehicle alone.

#### Behavioral protocols

*Y-maze.* The Y-maze test is a common method to evaluate cognitive changes in mice, including spatial working memory, measured by spontaneous alternation, and exploratory activity, assessed through the total number of arm choices. The Y-maze equipment is made up of a black, horizontal maze with 3 arms arranged ay angles of 120°, measuring 40 cm long, 10 cm wide, and 12 cm tall. To conduct the Y-maze test, the mice were placed at the end of one arm and allowed to move freely through the maze for 8 min. We documented the total number and sequence of arm choices made by each mouse. We calculated the percentage of alternation as the ratio of arm choices that were different from the previous 2 choices. To reduce the effect of olfactory cues, we sprayed the interior of the maze with a 70% ethanol solution before every trial. The formula for calculating the alternation percentage is as follows: Alternation percentage was calculated as the number of alternations divided by the total arm entries multiplied by 100%.

*Novel object recognition.* We conducted novel object recognition after the habituation phase. The mice were gently handled for 5 to 10 min, one per day, for 5 consecutive days to familiarize them with the experimenter. Afterward, the mice were brought to the test room once per day for 3 consecutive days for further habituation. During the object familiarization phase, we placed each mouse individually in an empty cage containing an object positioned at the center. This object was not used in the subsequent experiments. We allowed the mice to explore the object free for 10 min. The mice took part in the object recognition task on the day of the test. The mice were presented with 2 identical objects twice, with each session lasting 6 min, separated by a 2-min intertrial interval. During the test phase, the mice had the opportunity to explore a familiar object and a novel object placed in the cage for 3 min. The time spent exploring each object was accurately recorded. To determine the preference for the novel object, the presence for the novel object was calculated as follows: The percentage of novel object preference was calculated as follows: Novel object preference = (Novel object exploration time/Total exploration time) × 100%. Furthermore, a discrimination index was calculated in order to evaluate the level of discrimination between the novel and familiar objects. The formula for the discrimination index is as follows: The discrimination index was calculated using the following formula: Discrimination index = (Novel object exploration time − familiar object exploration time)/(Novel object exploration time + Familiar object exploration time) × 100%. To ensure unbiased results and eliminate olfactory cues, all behavioral apparatus and object used in the trials were cleaned with 70% ethanol between trials and prior to each test phase.

*Morris water maze test.* We conducted the Morris water maze test to assess spatial learning and memory in the mice. In the training phase, we submerged a transparent rescue platform approximately 0.5 to 1.0 cm below water’s surface at a fixed location within the pool. During the first training day, we allowed the mice to stand on the platform for 10 s to become familiar with it. After that, we put the mice into the water maze, facing the pool’s wall, and allowed them to explore freely for 1 min. If a mouse could not locate the platform during this period, we gently guided it to the platform. Following each training trail, the mice rested on the platform for 10 s before being retrained from a different starting position using the same procedure. This procedure was repeated for a total of 4 training trials in a day. The mice were trained for 5 consecutive days, with the exception of the initial habituation session. Following a 24-h rest period, a probe test was carried out. During the probe test, the platform was removed from the pool, and the mice swam in the maze for 1 min. The mice’s behavior was recorded and analyzed using Noldus EthoVision XT software (Noldus Information Technology, The Netherlands). The Morris water maze was divided into 4 quadrants virtually, with the initial platform location marked as the target quadrant. The evaluation of spatial learning and memory was based on measuring the escape latency, which refers to the time the mice took to find the platform during the 5-day training session. A shorter escape latency signifies superior spatial learning and memory. During the reverse learning phase, the platform was relocated to the opposite quadrant, and the identical training procedure was performed once more. This phase enabled us to evaluate behavioral flexibility under changes in the platform’s location.

*Open field test.* We used the open field test [76] to study how genetic manipulation of *Sh2b1* affects innate anxiety-like behaviors and locomotor responses in new environments. We conducted the test in a 40 × 40 × 35 cm square Plexiglas apparatus, which was under diffuse lighting. We divided the arena into 2 zones: the first, a “center” zone measuring 20 × 20 cm, and the second, a “corner” zone that occupied the rest of the area. The mice were given a minimum of 1 h to adapt to the testing environment before begging the actual test. The mice were positioned in the center of the square and given unrestricted access to explore the environment for 30 min during the testing session. The apparatus was thoroughly cleaned after each trial, and the mice were subsequently returned to their home cage. Truscan, a specialized system for detecting and monitoring mouse behavior in the open field test, was used to detect mouse activity and data recording.

*Elevated zero maze*. The elevated zero maze is a modified version of the plus maze that assesses 2 opposing innate tendencies: the drive to explore a novel environment and the natural aversion to elevated and open spaces, which are perceived as risky due to the potential for predation. This test aims to evaluate these tendencies objectively. The apparatus consists of a circular pattern with 2 open (stressful) and 2 closed (protective) elevated arms. This arrangement resembles a zero or circle shape. The apparatus aims to objectively evaluate the natural aversion and drive to explore in mice. Mice are placed at the center of the maze and allowed to explore the maze freely for 5 min during the test. This test allows for establishing the level of anxiety in mice by measuring the time they explore the closed versus open arms. To record and measure the mice, a digital camera was installed directly above the maze, capturing images at a frequency of 5 Hz. We used the ANY-maze video tracking system (Stoelting Co., USA) to analyze and interpret the data. The mice were given at least 1 h to acclimate to the testing room before the actual testing.

#### Metabolic measurement

Male mice that were 8 weeks old underwent metabolic assessments, which included measurements of oxygen consumption (VO_2_), carbon dioxide production (VCO_2_), respiratory exchange ratio, physical activity, and energy expenditure. The Promethion Metabolic Screening System (FG250; Sable Systems International, North Las Vegas, NV) was used to conduct these measurements, following the manufacturer’s guidelines. The data were analyzed using calR templates.

#### Glucose tolerance test

To conduct the GTT, mice fasted overnight for approximately 16 h, and their fasting blood glucose levels were measured. Then, glucose was intraperitoneally administered, and blood glucose levels were measured 15, 30, 60, 90, and 120 min after the injection during the test.

#### Insulin tolerance test

Initially, mice fasted for approximately 4 h and their blood glucose levels were determined before conducting the ITT. Afterward, insulin was intraperitoneally administered, and blood glucose levels were measured at 15, 30, 60, 90, and 120 min after injection during the test.

#### MRI measurements

MRI measurements were performed using a Bruker BioSpec 11.7T scanner with a CryoProbe from Ettlingen, Germany. The scanner was controlled by means of the Bruker Paravision 6.0.1 software. Axial and sagittal multi-slice TurboRARE T2-weighted images were obtained with the parameters below: Axial direction: The MRI scans were performed with a repetition time (TR) of 3,000 ms and an echo time (TE) of 12 ms, using a rare factor of 8 for signal acquisition. The acquired data consisted of one average and 20 slices, with a field of view (FOV) of 19.2 mm × 19.2 mm and a matrix of 256 × 256. The scans were performed in the sagittal plane. The MRI scans were performed with a TR of 3,000 ms and a TE of 10 ms, using a rare factor of 8 for signal acquisition. The acquired data consisted of one average and 13 slices, with an FOV of 19.2 mm ×12.8 mm and a matrix of 128 × 128. The respiratory rate of the mice was continuously monitored during the MRI scans using a small animal monitoring system (SA Instruments, Stony Brook, NY, USA). The concentration of isoflurane (0.5% to 1%) in oxygen was adjusted to maintain a respiratory rate below 60 breaths per minute. During the MRI scans, the body temperature of the mice was monitored using a rectal sensor and regulated by controlling the temperature of the water flowing through tubes covering the body of each mouse, maintaining it at 18–22°C. Custom scripts in MATLAB (MathWorks, Natick, MA) and SPM12 software (http://www.fil.ion.ucl.ac.uk/spm/) were used to process the acquired MRI data. ITK-SNAP software (http://www.itksnap.org/) was used for manual brain extraction after image format conversion. For analysis, the Turone Mouse Brain Atlas and Template, a high-resolution mouse structural template, was selected [77]. We conducted a 2-sample *t* test and created visual representations using DPABI [78]. We used a statistical significance level of *P* < 0.05 for the analysis.

#### Western blotting

Western blotting was conducted in adherence to established protocols [79]. The hippocampus was quickly dissected and homogenized in radioimmunoprecipitation assay buffer, consisting of protease and phosphatase inhibitors [25 mM Tris–HCl, pH 7.6, 150 mM NaCl, 1 mM EDTA, 1% (v/v) NP-40, 0.5% (w/v) sodium deoxycholate, and 0.1% (w/v) SDS], following established procedures. A bicinchoninic acid assay was employed to determine protein concentration, after which each sample (40 μg) was denatured in Laemmli buffer, boiled for 10 min, resolved by 4% to 15% SDS-PAGE and transferred to a nitrocellulose membrane. The membranes were blocked with 5% (w/v) nonfat milk in Tris-buffered saline/0.1% (v/v) Tween 20 (TBST) for 1 h and then incubated overnight at 4 °C with primary antibodies against α-tubulin (1:1,000; Proteintech Group, USA; catalog no. 11224-1-AP), Sh2B1 (1:1,000; R&D Systems, USA; catalog no. AF6915), phosphorylated ERK1/2 (1:1,000; Cell Signaling Technology, USA; catalog no. 4370), ERK1/2 (1:1,000; Proteintech Group, USA; catalog no. 66192), phospho-MEK1/2 (1:1,000; Cell Signaling Technology, USA; catalog no. 9122), MEK1/2 (1:1,000; Cell Signaling Technology, USA; catalog no. 9154), phospho-P38 MAPK (1:1,000; Cell Signaling Technology, USA; catalog no. 4511), P38 (1:1,000; Cell Signaling Technology, USA; catalog no. 8690), phospho-SAPK/JNK (1:1,000; Cell Signaling Technology, USA; catalog no. 4668), and SAPK/JNK (1:1,000; Cell Signaling Technology, USA; catalog no. 9252). The membranes were washed extensively with TBST and then incubated with horseradish peroxidase-conjugated secondary antibodies. The membranes were visualized using Enhanced Chemiluminescence (ECL) Plus (Beyotime Biotechnology, China). After washing in TBST, the membranes were stripped and reprobed with the appropriate total protein-specific antibody. The optical densities of the Immunoreactive bands were measured using the NIH ImageJ 1.63 program (NIH, USA). The Sh2B1 optical density values were normalized to those of α-tubulin. The results were expressed as a percentage compared to the control group.

#### Single-molecule fluorescence in situ hybridization

Fresh frozen sections were used to conduct single-molecule fluorescence in situ hybridization by means of RNAScope Multiplex Reagent Kits (ACDBio, USA). The sections were briefly cut to 20 μm thickness and air-dried in a cryostat for less than 20 min. After that, the frozen sections were fixed in ice-cold 4% paraformaldehyde (PFA) for 15 min and dehydrated using an ethanol series. The sections were then treated with H_2_O_2_ for 10 min and washed with PBS. To initiate hybridization, RNA probes such as VGLUT1, VGAT, and Cre were incubated with the sections in a HybEZ humidified incubator set at 40 °C for 2.5 h. After incubation, the sections were cleaned using ACD wash buffer and then subjected to sequential incubation with AMP1-FL and AMP2-FL reagents for 30 min each, now followed by incubation with AMP3-FL for 15 min. The last step involved staining the sections with diamidino-2-phenylindole (DAPI) to enable visualization of the cell nuclei. The fluorescent signals from the labeled RNA probes and DAPI-stained cell nuclei were observed using an Olympus FV3000 confocal system for image acquisition.

#### Isolation of nuclei from mouse hippocampal tissue for 10x genomics single-cell RNA sequencing

Nuclei were isolated from mouse hippocampal tissue for single-cell RNA sequencing with the 10x Genomics method, using the following protocol: The hippocampus of either *Sh2b^ΔIN^* or *Sh2b^IN-Ctrl^* mice was manually dissociated and then isolated. Following the 10x Genomics protocol for “Nuclei Isolation from Cell Suspensions & Tissues for Single Cell RNA Sequencing”, nuclei were isolated from the mouse hippocampal region. The preparation of single-nucleus RNA sequencing libraries was performed using the Chromium Single Cell 30 v2 Library kit (10x Genomics) [80] as per the manufacturer’s instructions. Around 10,000 nuclei per sample were loaded for further processing. The reverse transcription and amplification steps were processed using a Bio-Rad T100 thermal cycler. Raw sequencing data generated from the *Sh2b^ΔIN^* and *Sh2b^IN-Ctrl^* hippocampal tissues were preprocessed using the Cell Ranger software (v2.1.1, 10x Genomics, Pleasanton, USA). The nucleotide sequences were mapped to the GRCm38 mouse genome, generating a feature-barcode matrix. The Seurat v2.0 package [81] was employed to perform the analysis of the feature-barcode matrix. Normalization was done by logarithmically scaling the filtered matrix to 10,000 transcripts per cell. Identification of variable genes across the cells was achieved via the application of the FindVariableGenes function. Unwanted sources of variation were eliminated. We generated dot plots and UMAP plots to display marker gene transcript abundance using the ggplot2 package. Furthermore, we created violin plots using the VlnPlot function to visualize marker gene expression across all clusters.

#### Quantification and statistical analysis

The sample sizes for animal experiments were not predetermined statistically but were selected based on the standard sizes used in the field. The data were randomly collected and processed, and all behavioral tests and analyses were conducted in a blinded manner. We tested the normality of data distributions and assessed the equality of variance between groups using Levene’s test. All data points were included in the analysis. Typically, histograms exhibit individual points and the sample counts for each condition. The data are reported as the mean ± SEM. The statistical significance was assessed using the appropriate test, such as 2-tailed Student's *t* test, 1-way analysis of variance (ANOVA), or 2-way repeated-measures ANOVA. When necessary, post-hoc comparisons were conducted using Fisher's least significant difference test. GraphPad Prism software was used for all statistical analyses, considering a statistical significance level of *P <* 0.05. Significance is indicated as *P < 0.05, **P < 0.01, and ***P < 0.001. In some instances of multiple comparisons, significance is denoted as ^#^*P <* 0.05, ^##^*P <* 0.01, and ^###^*P <* 0.001. Non-significant values are not explicitly indicated unless required for emphasis.

## Data Availability

All the data needed to evaluate the conclusions in the paper are present in the paper and in the Supplemental Materials. Additional data related to this paper may be requested from the authors.
