# Supplementary material for: SH2B1 Tunes Hippocampal ERK Signaling to Influence Fluid Intelligence in Humans and Mice
Source: Research (Wash D C). 2023 Nov 14;6:0269. doi: 10.34133/research.0269 (PMC10907025; doi:10.34133/research.0269)
Supplement: Supplementary 1 — Figs. S1 to S21 Tables S1 to S15 [file research.0269.f1.zip › Supplemental Material_Sh2b1-Fluid intelligence_20231019.docx]

Supplemental material for

**SH2B1 tunes hippocampal ERK signaling to influence fluid intelligence in humans and mice**

Xiujuan Du^1-4,^^†^, Yuhua Yan^1,2,5,6,†^, Juehua Yu^1,2,7,†^, Tailin Zhu^1,2,5,9^, Chu-Chung Huang^8^, Lingli Zhang^1,2^, Xingyue Shan^1,2,5^, Ren Li^3,4^, Yuan Dai^1,2^, Hui Lv^1,2^, Xiao-Yong Zhang^3,4^, Jianfeng Feng^3,4^, Wei-Guang Li^6,*^, Qiang Luo^3,4,*^, Fei Li^1,2,9*^

The file includes:

Fig. S1 to S21

**
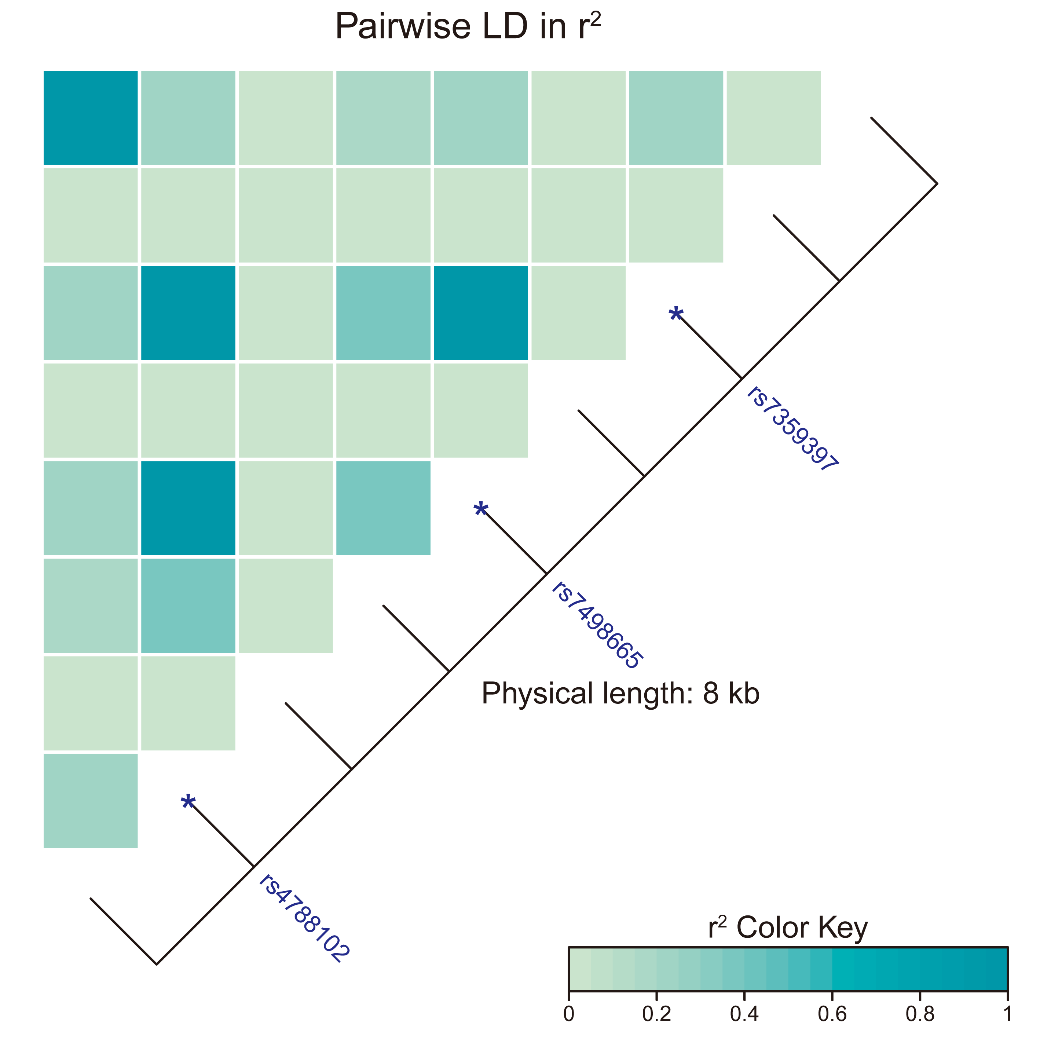
**

**Fig. S1. Pairwise linkage disequilibrium (LD) plot of SNPs within the *SH2B1* gene**. LD plots were generated based on *r*^2^ values. Three SNPs (rs4788102, rs7359397, and rs7498665) significantly associated with intelligence were in high LD with each other.

**
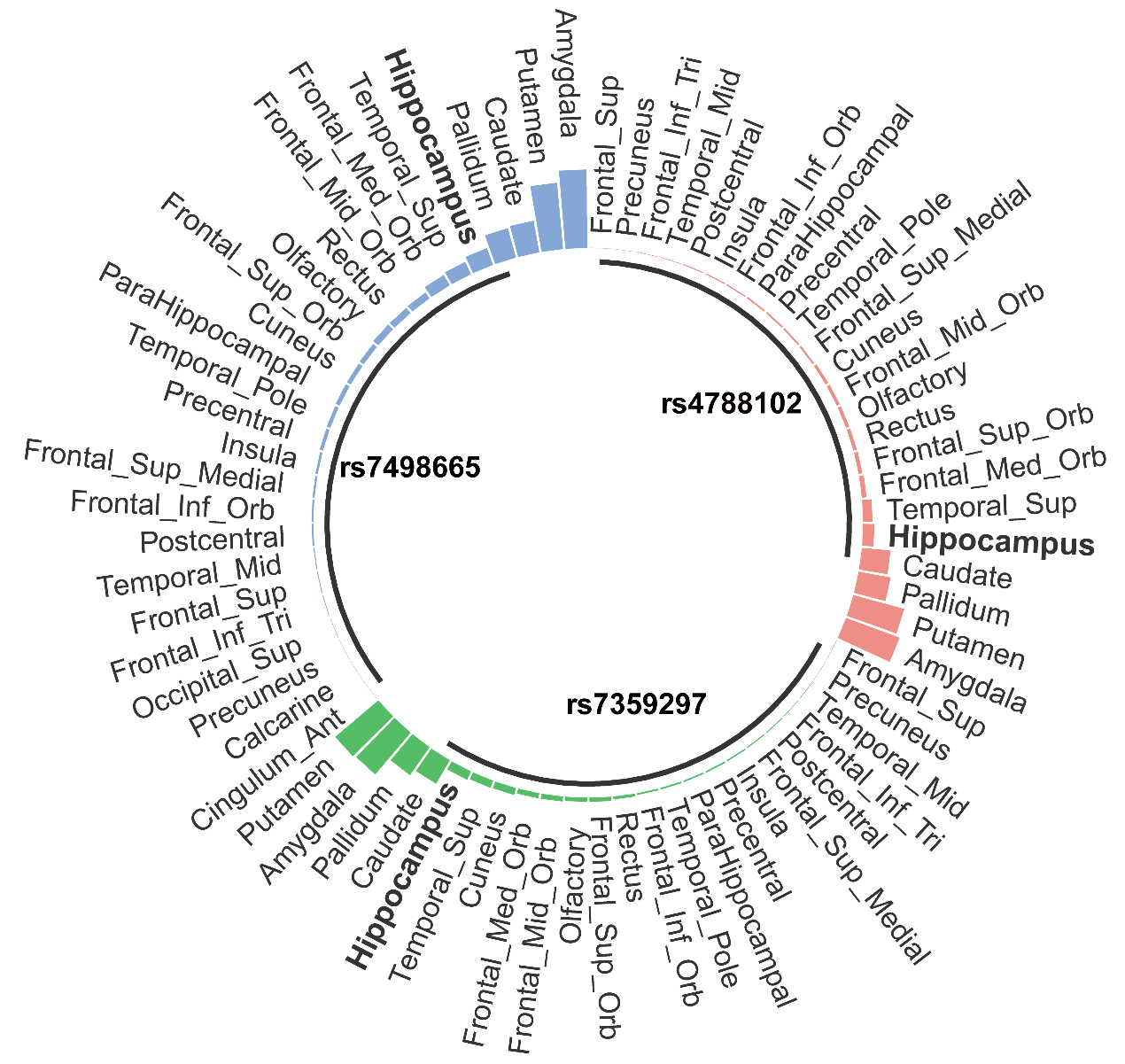
**

**Fig. S2. The relationship between 3 SNPs within *SH2B1* and the brain regions of interest based on voxel-wise analysis.**

**
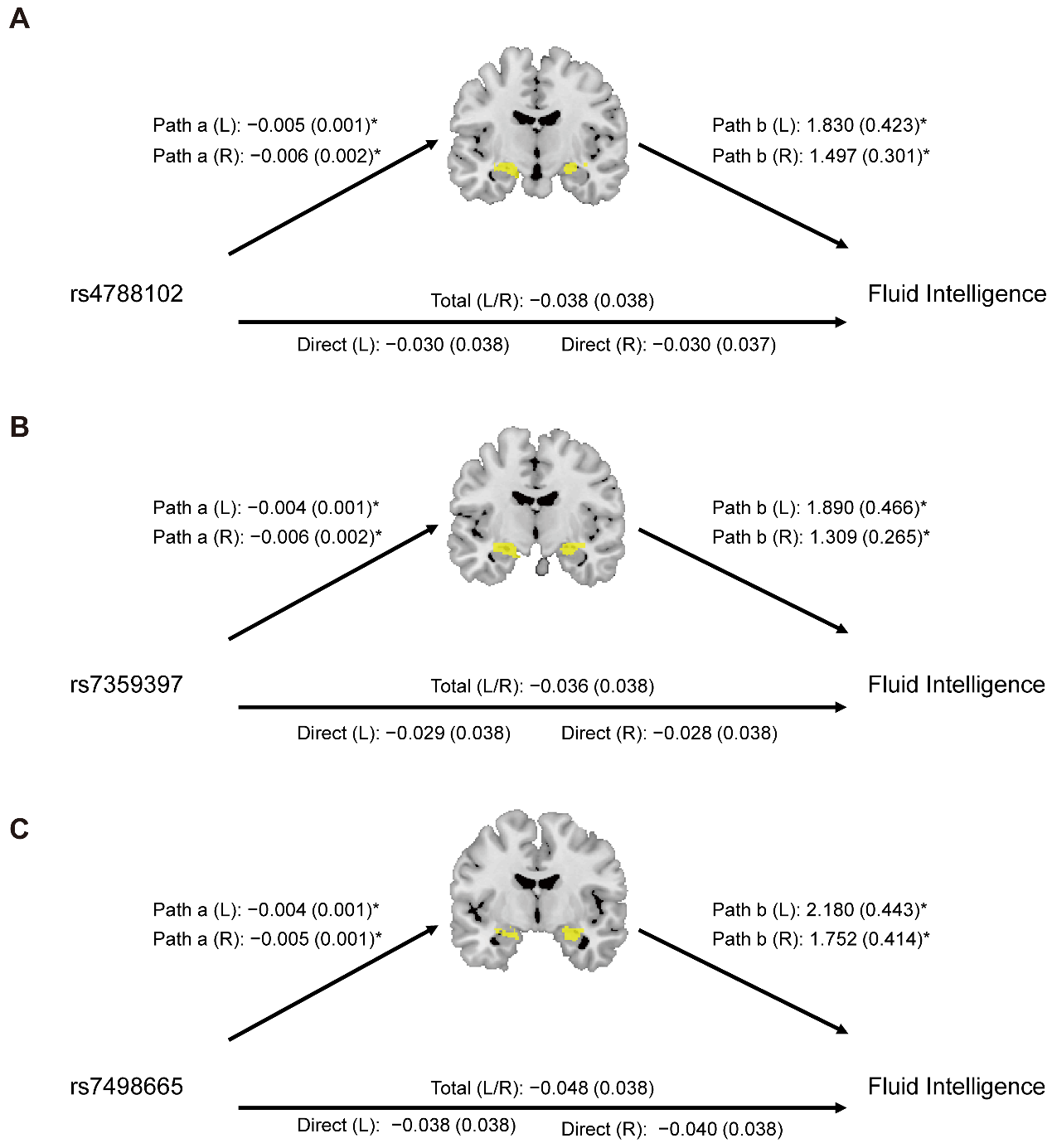
**

**Fig. S3. Mediation results for rs4788102, rs7359397, and rs7498665 in *SH2B1* in the bilateral hippocampi (with BMI not controlled for in the analysis).** The path diagram shows the mediation model adjusted for age at the imaging visit, sex, the 15 PCs, and eTIV. Significant regions (*p <* 0.005, two-tailed, and 3 contiguous voxels in each of paths a, b, and a*b) mediating the correlation between three SNPs (**A**, rs4788102; **B**, rs7359397; **C**, rs7498665) and the fluid intelligence scores. **p <* 0.001, two-tailed, 1,000 bootstraps.


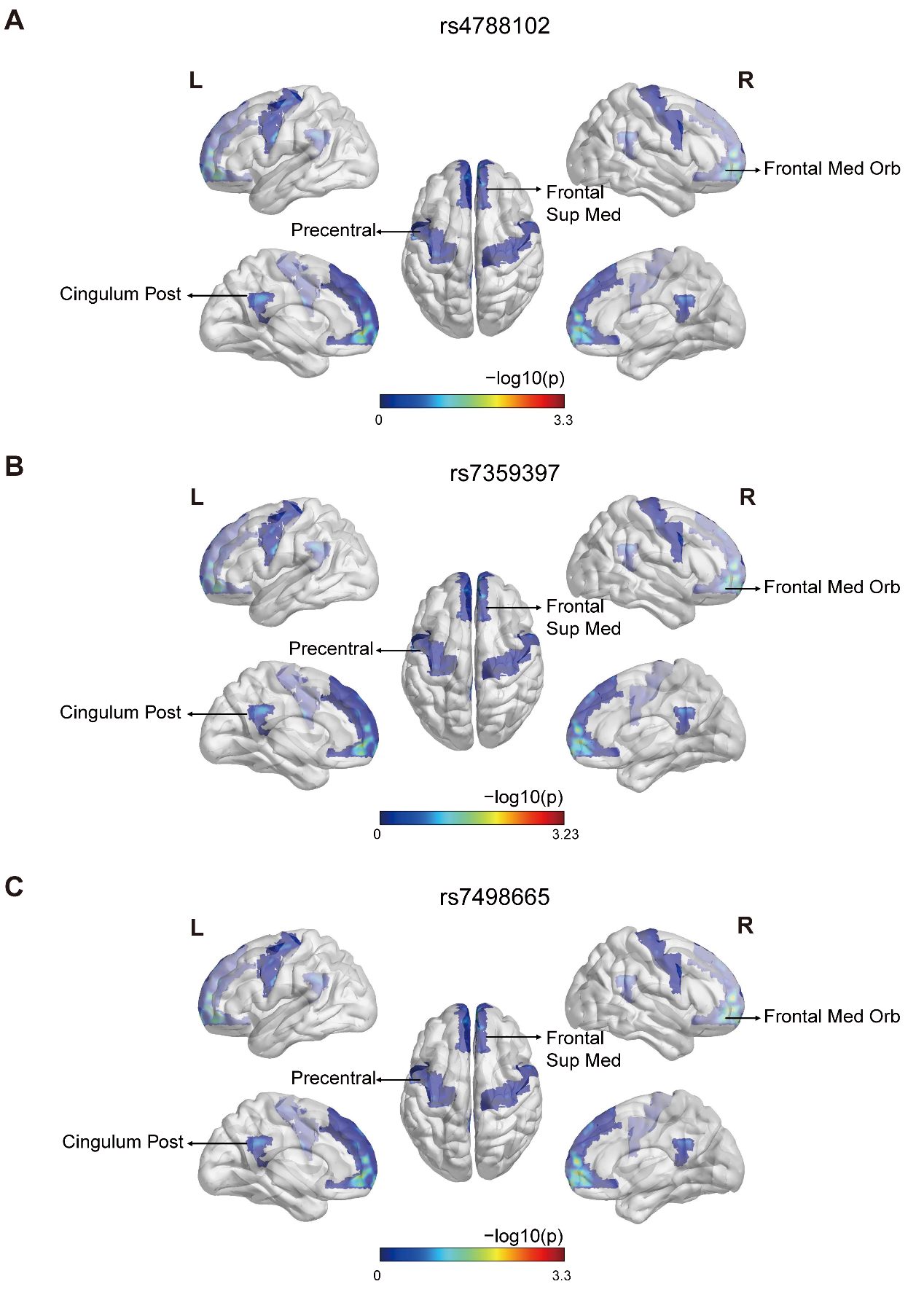


**Fig. S4. Mediation effects of brain volumes on the association between SNPs within *SH2B1* (rs4788102, rs7359397, and rs7498665) and the FI.** In the mediation analyses, age at the imaging visit, sex, the top 15 principal coordinates (PCs), and the estimated total cranial volume (eTIV) were controlled as covariates. −log10(p) of path a*b mediating the correlation between three SNPs and fluid intelligence scores were shown in the figure. Frontal Sup Med, frontal superior medial cortex; Frontal Med Orb, frontal medial orbital cortex; Cingulum Post, posterior cingulate cortex; Precentral, precentral gyrus cortex; L, left; R, right.


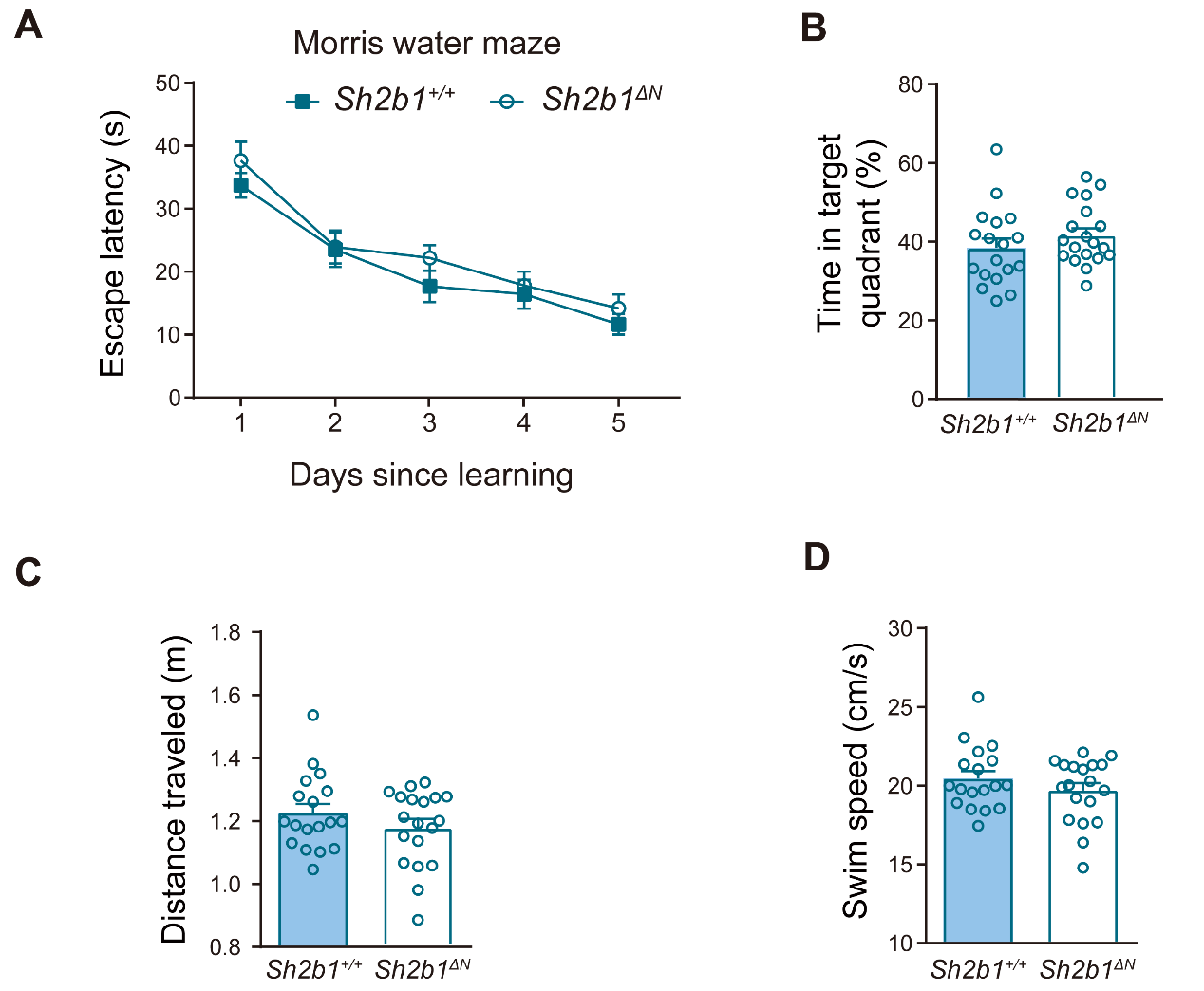


**Fig. S5. The effects of *Sh2b1* gene deletion on the behaviors of mice in the Morris water maze test.** Data are shown as means ± S.E.M. *Sh2b1^+/+^*, *n* = 18; the mice with hippocampal neuron-specific knockout of *Sh2b1* (*Sh2b1^ΔΝ^*), *n* = 19. (**A**) Learning curve during spatial training in the hidden platform of the Morris water maze. The latency for each mouse to reach the hidden platform was recorded. Data are shown as the mean ± S.E.M. Two-way repeated measures ANOVA, main effect of group, F_1,19_ = 2.875, p = 0.1063. *p* = 0.1573, 0.9315, 0.1728, 0.5861, and 0.2964, from the first to the fifth day since learning, *Sh2b1^+/+^* versus *Sh2b1^ΔΝ^*, unpaired Student’s *t*-test. (**B**−**D**) Average time spent in the target quadrant (**B**), total distance swam (**C**), and swimming speed (**D**) during probe trials. *p* = 0.2796, 0.2503, and 0.2592, for time in target quadrant (**B**), distance traveled (**C**), swim speed (**D**), respectively, *Sh2b1^+/+^* versus *Sh2b1^ΔΝ^*, unpaired Student’s *t*-test.


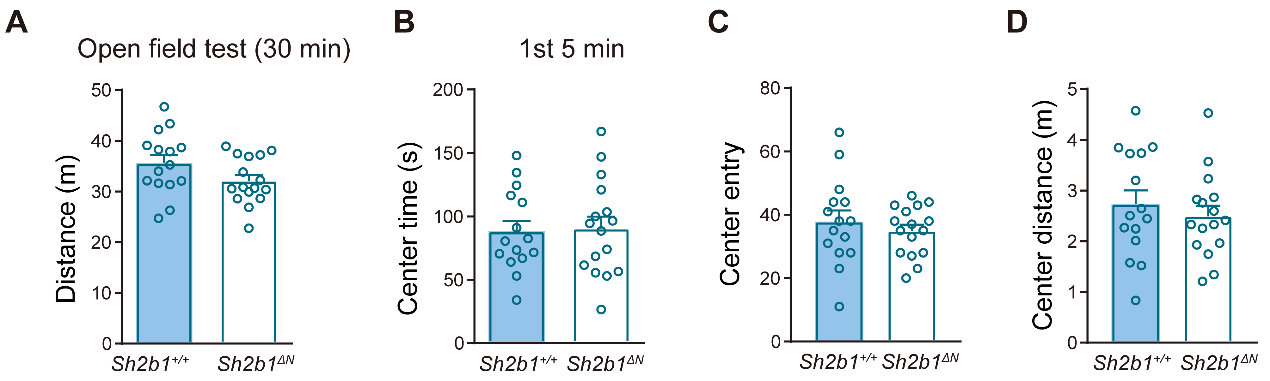


**Fig. S6. The effects of *Sh2b1* gene deletion on the behaviors of mice in the open field test.** Data are shown as means ± S.E.M. *Sh2b1^+/+^*, *n* = 15; the mice with hippocampal neuron-specific knockout of *Sh2b1* (*Sh2b1^ΔΝ^*), *n* = 16. (**A**) Total distance traveled in 30 min in the open field test. (**B**) Time spent in the central area during the first 5-min test. (**C**) The number of mice that entered the center area during the first 5-min test. (**D**) Distance traveled in the center area during the first 5-min test. *p* = 0.0846, 0.8589, 0.4783, and 0.4786, for distance (**A**), center time (**B**), center entry (**C**), and center distance (**D**), respectively, *Sh2b1^+/+^* versus *Sh2b1^ΔΝ^*, unpaired Student’s *t*-test.

**
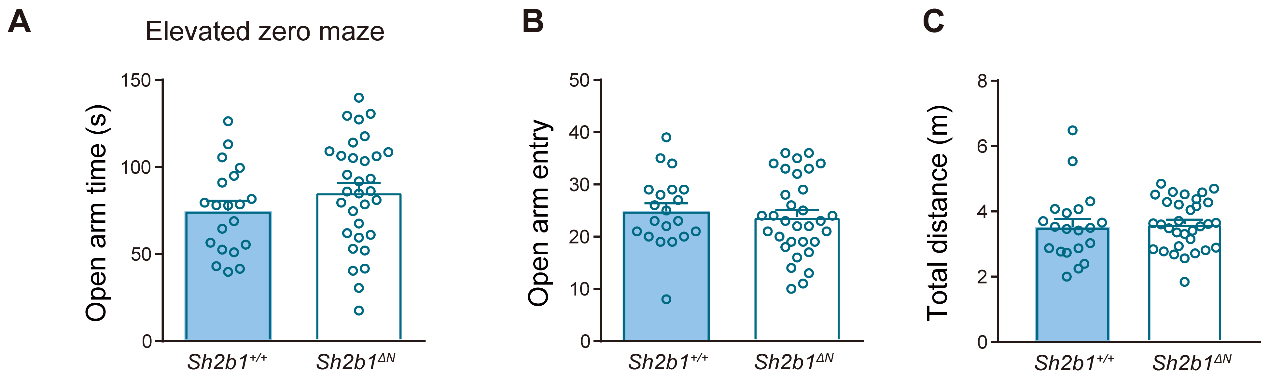
**

**Fig. S7. The effects of *Sh2b1* gene deletion on the behaviors of mice in the elevated zero maze test.** *Sh2b1^+/+^*, *n* = 20; the mice with hippocampal neuron-specific knockout of *Sh2b1* (*Sh2b1^ΔΝ^*), *n* = 32. (**A**) Time spent in an open arm in the 5 min test. (**B**) The number of mice that entered an open arm. (**C**) Total distance traveled. *p* = 0.2060, 0.6076, and 0.7453, for open arm time (**A**), open arm entry (**B**), and total distance (**C**), respectively, *Sh2b1^+/+^* versus *Sh2b1^ΔΝ^*, unpaired Student’s *t*-test.

**
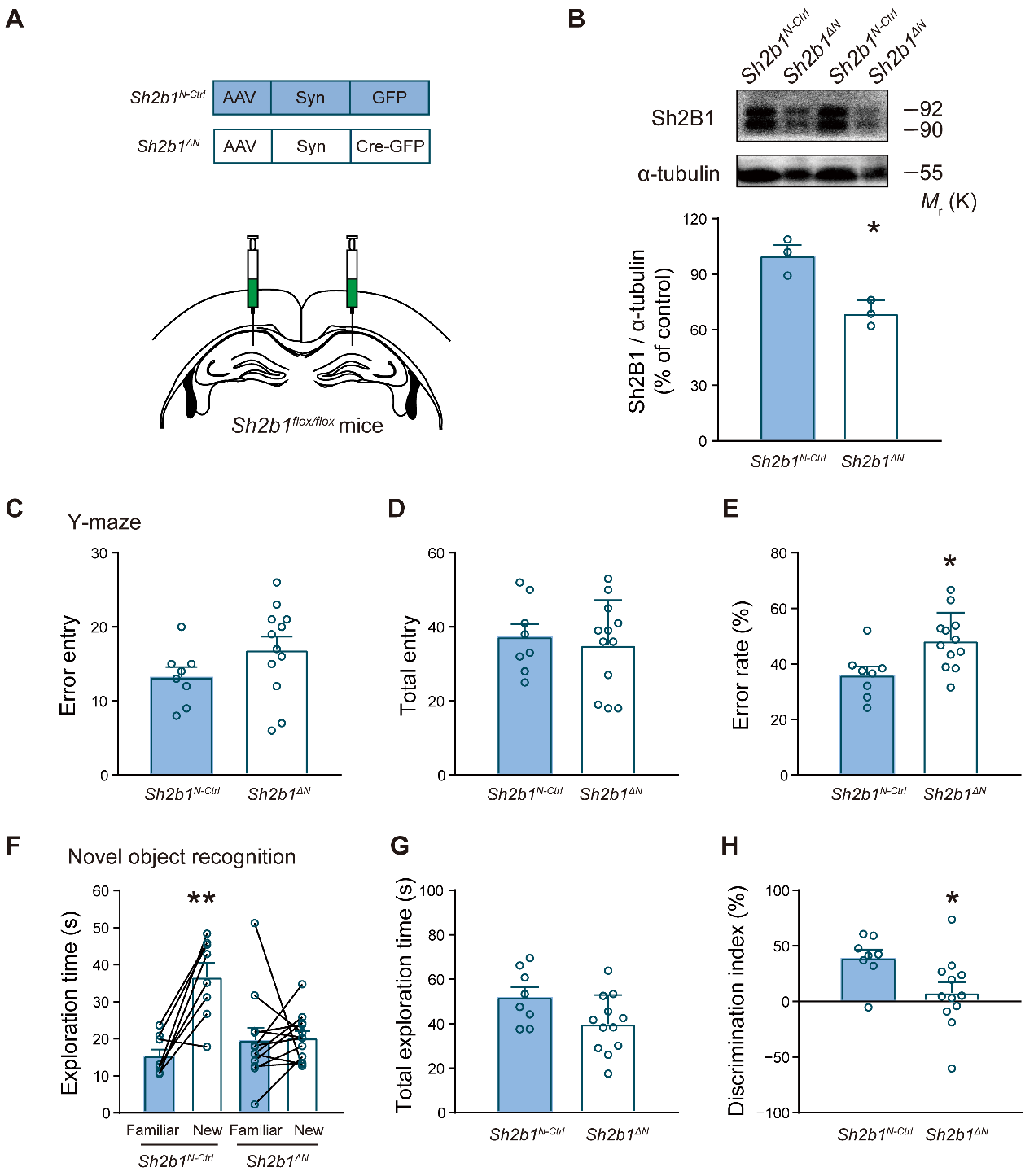
**

**Fig. S8. The effects of hippocampal neuron-specific *Sh2b1* gene deletion on intelligence-related behaviors were investigated using AAV-Syn-GFP or AAV-Syn-Cre-GFP injected into the *Sh2b1^flox/flox^* mice, respectively**. (**A**) Schematics of AAV injections into the *Sh2b1^flox/flox^* mice to obtain mice null for *Sh2b1* selectively in hippocampal neurons (*Sh2b1^ΔΝ^*, while *Sh2b1^Ν-Ctrl^* as the control group). (**B**) Representative immunoblots and pooled data show that AAV-Cre reduced Sh2B1 protein expression in hippocampal CA1 regions. The immunoreactivity of Sh2B1 was normalized to that of the total protein and is expressed as percentage of the control group (AAV-GFP injection to *Sh2b1^flox/flox^* mice). Data are shown as means ± S.E.M. *Sh2b1^N-Ctrl^*, n = 3; the mice with hippocampal neuron-specific knockout of *Sh2b1* (*Sh2b1^ΔΝ^*), *n* = 3. *p* = 0.0114 (*), unpaired Student’s *t*-test. (**C**−**E**) Test of Y-maze. Error entries (**C**), total entries (**D**), and error rate (%, **E**) are shown as means ± S.E.M. *Sh2b1^N-Ctrl^*, n = 8; *Sh2b1^ΔΝ^*, *n* = 12. *p* = 0.1495, 0.6617, and 0.0107 (*), for error entries (**C**), total entries (**D**), and error rate (%, **E**), *Sh2b1^+/+^* versus *Sh2b1^ΔΝ^*, unpaired Student’s *t*-test. (**F** to **H**) Exploration time for each object (**F**), total exploration time (**G**), discrimination index over the novel object (**H**) are shown as means ± S.E.M. *Sh2b1^N-Ctrl^*, n = 8; *Sh2b1^ΔΝ^*, *n* =12. (**F**) *Left*, *Sh2b1^N-Ctrl^*, *p* = 0.0012 (**); *Right*, *Sh2b1^ΔΝ^*, *p* = 0.8756, Familiar versus New, paired Student’s *t*-test. (**G**) Total exploration time, *p* = 0.0518, *Sh2b1^N-Ctrl^* versus *Sh2b1^ΔΝ^*. (**H**) Discrimination index, *p* = 0.0264 (*), *Sh2b1^N-Ctrl^* versus *Sh2b1^ΔΝ^*.

**
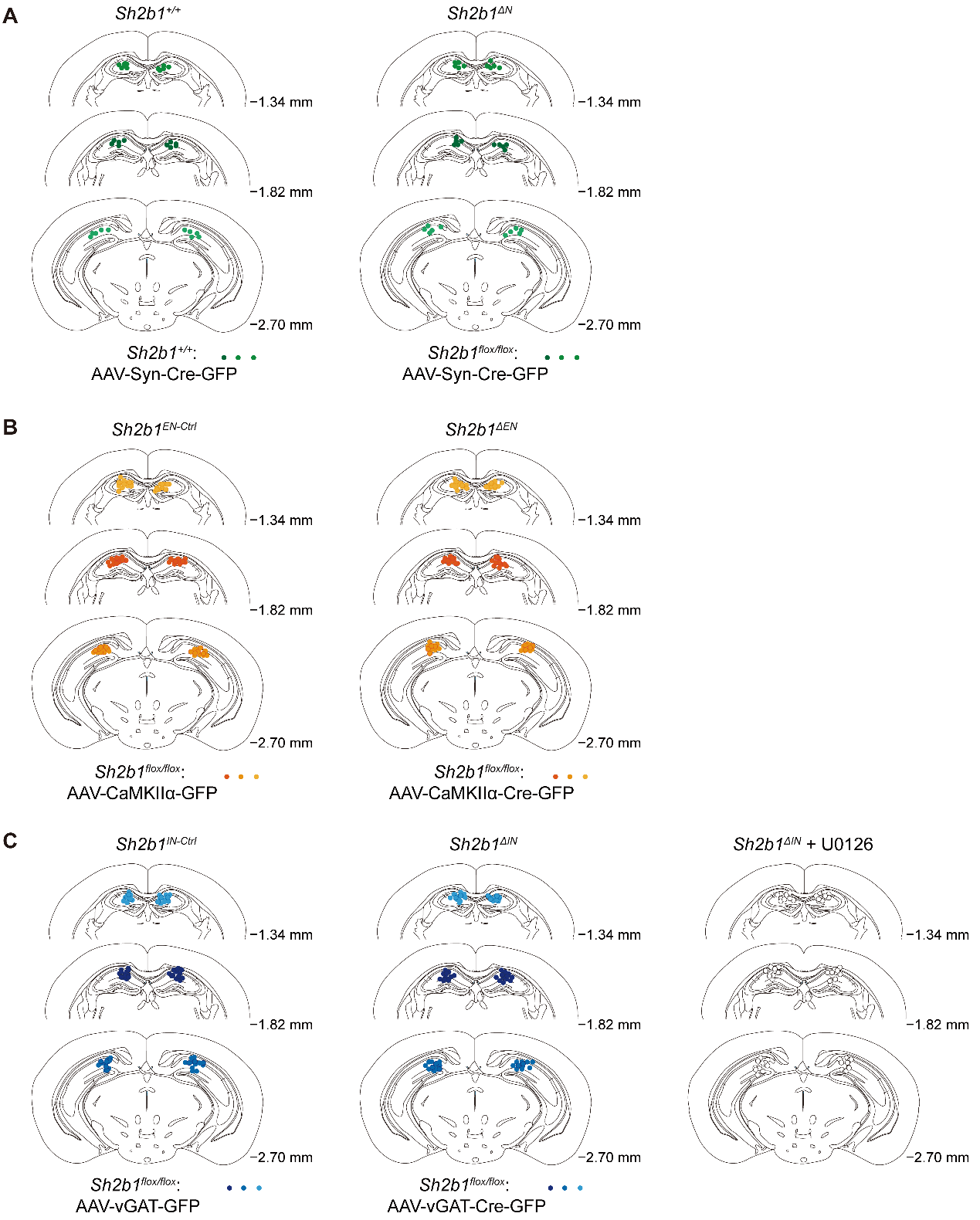
**

**Fig. S9. Histological confirmation of viral expression and cannula placement.** (**A**) *Sh2b1^+/+^* and *Sh2b1^ΔΝ^* mice shown in Figure 5. (**B**) *Sh2b1^EN-Ctrl^* and *Sh2b1 ^ΔΕΝ^* mice for the data shown in Figure 6. (**C**) Left, *Sh2b1^IN-Ctrl^* and *Sh2b1 ^ΔIN^* mice for the data shown in Figures 6 and 8. Right, histological verification of cannula placement for the data shown in Figure 8.

**
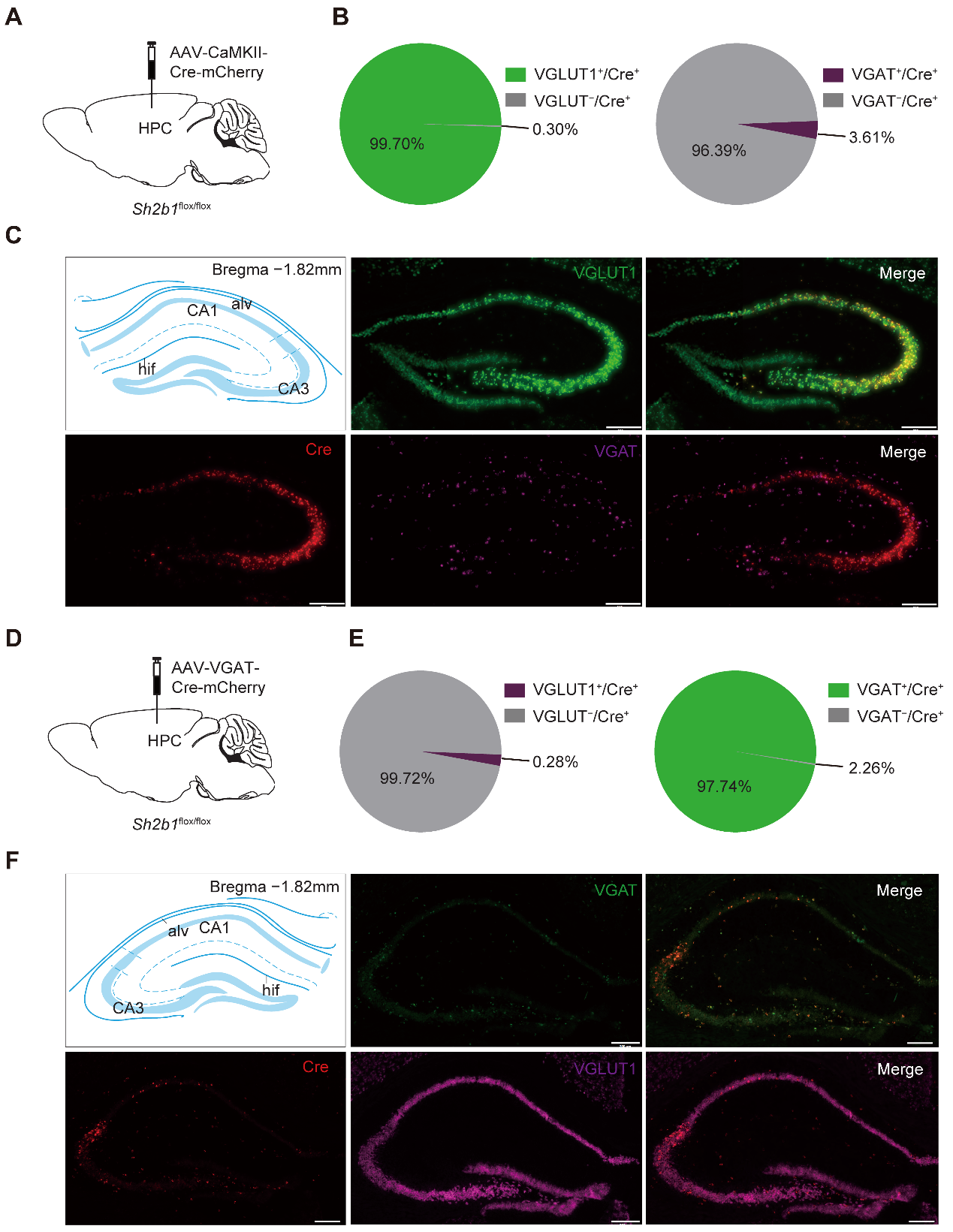
**

**Fig. S10. Histological verification of viral infection specificity using RNAscope.** (**A**, **D**) Schematics of AAV injections. (**B**, **C**) Representative photographs and quantification (pie chart) show large double staining of VGluT1 and Cre but minimal double staining of VGAT and Cre in the hippocampus using AAV vector under the control of CaMKIIα promoter. n = 4 sections per mouse from 6 mice. (**E**, **F**) Representative photographs and quantification (pie chart) show large double staining of VGluT1 and Cre but minimal double staining of VGAT and Cre in the hippocampus using AAV vector under the control of VGAT promoter. n = 4 sections per mouse from 8 mice.

Scale bars, 200 μm.

**
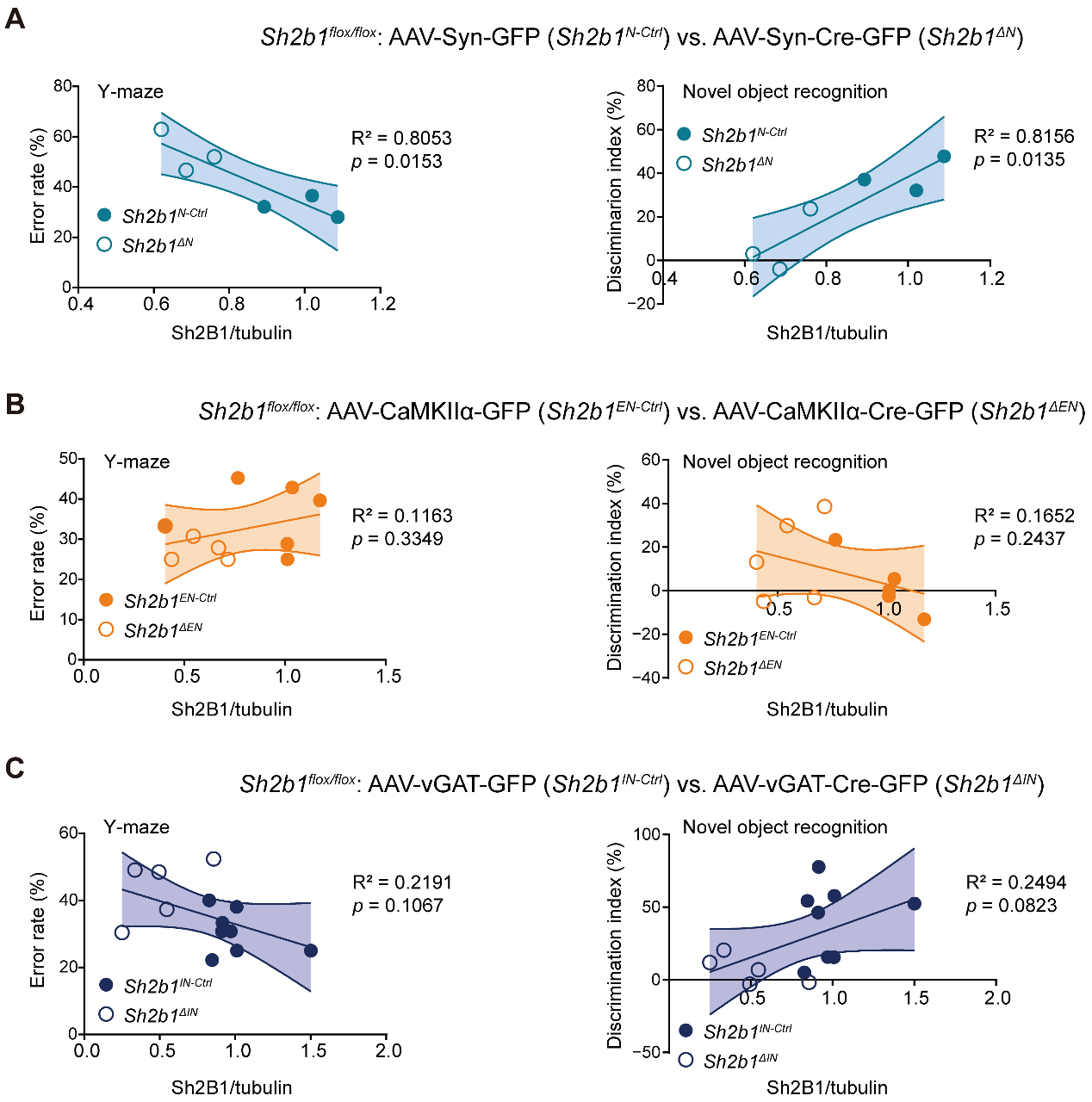
**

**Fig. S11. Correlation between intelligence-related behaviors in mice and the relative expression of hippocampal Sh2B1 expression**. Left, Correlation of the error rate in the Y-maze and the relative expression of Sh2B1. Right, Correlation of the discrimination index in the novel object recognition test and the relative expression of Sh2B1. (**A**) Data are from Fig. S8. *Sh2b1^N-Ctrl^* and *Sh2b1^ΔΝ^*. (**B**) Data are from Figure 6. *Sh2b1^EN-Ctrl^* and *Sh2b1^ΔEΝ^*. (**C**) Data are from Figure 6. *Sh2b1^IN-Ctrl^* and *Sh2b1^ΔIΝ^*. Pearson’s coefficient (R) and *p*-values are given for each plot.


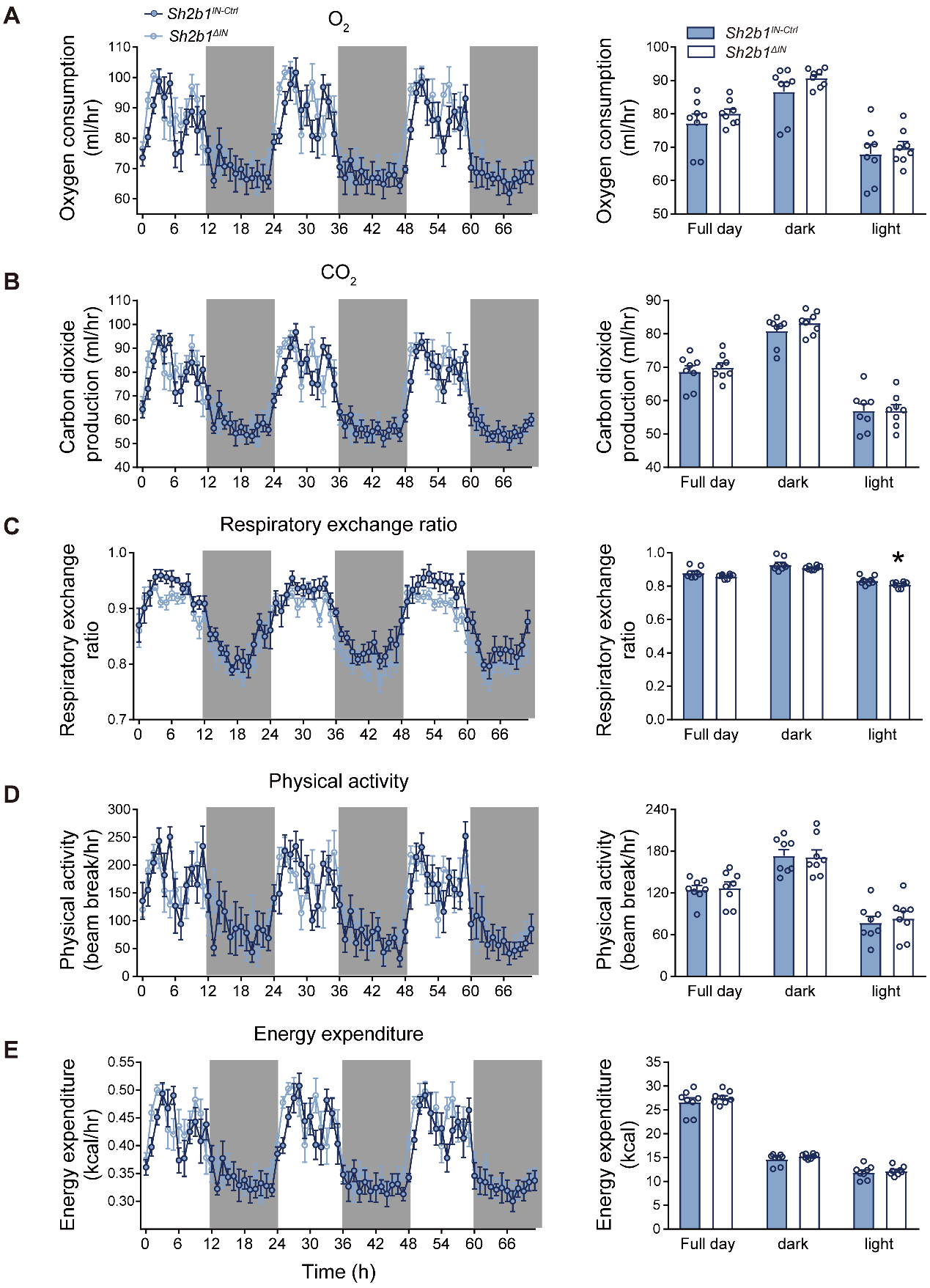


**Fig. S12. Effects of hippocampal interneuron-specific *Sh2b1* gene deletion on the basic metabolic parameters.** *Left*, oxygen consumption (**A**), carbondioxide production (**B**), respiratory exchange ratio (**C**), physical activity (**D**), and energy expenditure (**E**) *per* hour. *Right*, timeslot statistics. *Sh2b1^IΝ-Ctrl^*, n = 8; *Sh2b1^ΔIΝ^*, n = 8. *Left*, two-way repeated measures ANOVA, main effect of group, (**A**) F_1,14_ = 0.8308, *p* = 0.3775; (**B**) F_1,14_ = 0.2341, *p* = 0.6360; (**C**) F_1,14_ = 3.64, *p* = 0.0771; (**D**) F_1,14_ = 0.0357, *p* = 0.8529; (**E**) F_1,14_ = 0.7126, *p* = 0.4128. *Right*, (**A**) *p* = 0.3519, 0.1800, and 0.6041, for full day, dark, and light, respectively; (**B**) *p* = 0.5778, 0.2358, and 0.9709, for full day, dark, and light, respectively; (**C**) *p* = 0.0817, 0.1902, and 0.0392 (*), for full day, dark, and light, respectively; (**D**) *p* = 0.8129, 0.9052, and 0.6426, for full day, dark, and light, respectively; (**E**) *p* = 0.3826, 0.1846, and 0.6640, for full day, dark, and light, respectively, *Sh2b1^IΝ-Ctrl^* versus *Sh2b1^ΔIΝ^*, unpaired Student’s *t*-test.


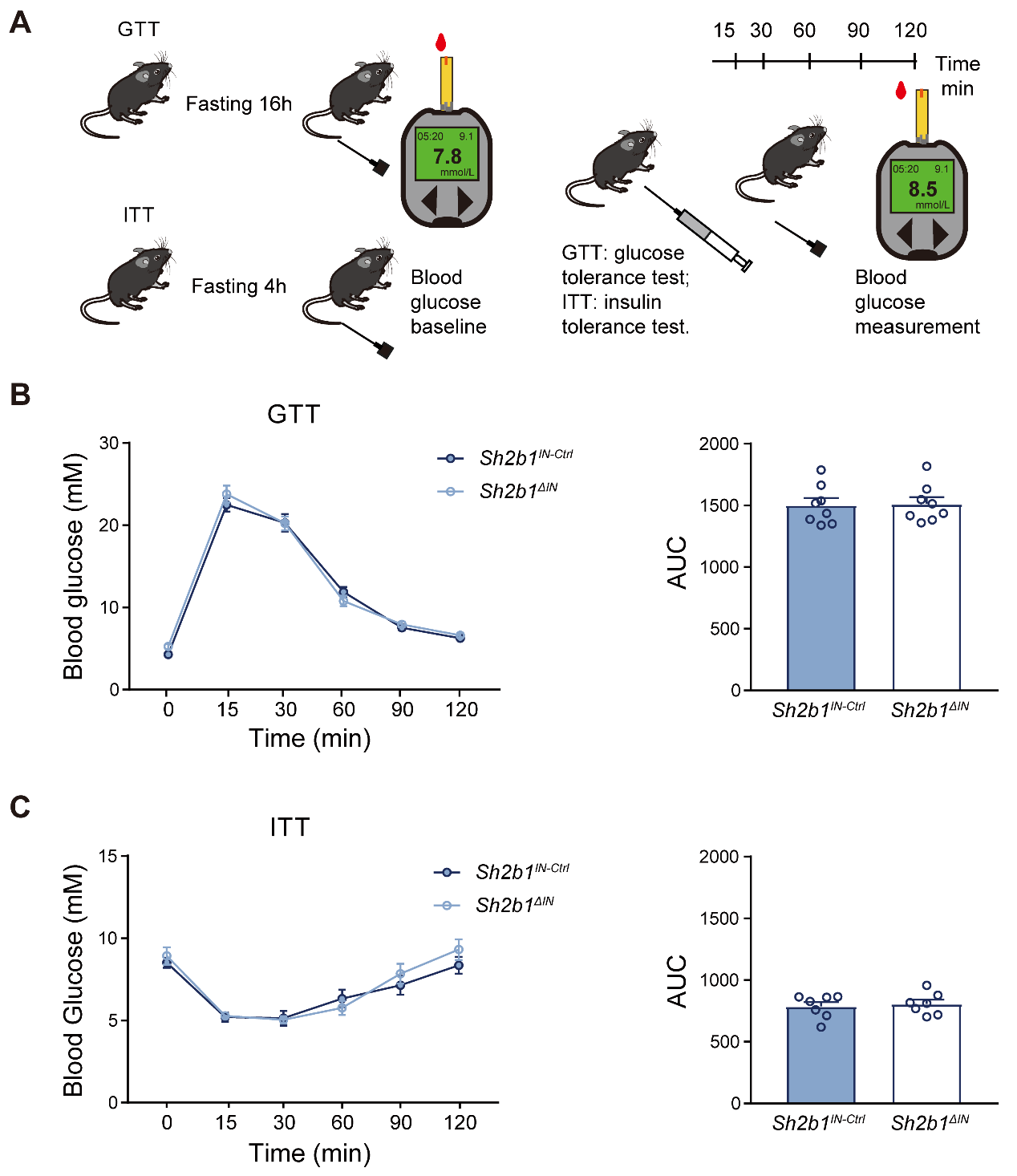


**Fig. S13. Effects of hippocampal interneuron-specific *Sh2b1* gene deletion on the glucose tolerance test (GTT) and the insulin tolerance test (ITT).** (**A**) Experimental scheme of GTT and ITT. (**B**) Glucose tolerance test data. n = 8 for each group. Two-way repeated measures ANOVA, main effect of group, F_5,42_ = 1.076, *p* = 0.3875. (**C**) Insulin tolerance test data. n = 7 for each group. Two-way repeated measures ANOVA, main effect of group, (A) F_5,70_ = 1.33, *p* = 0.2618.


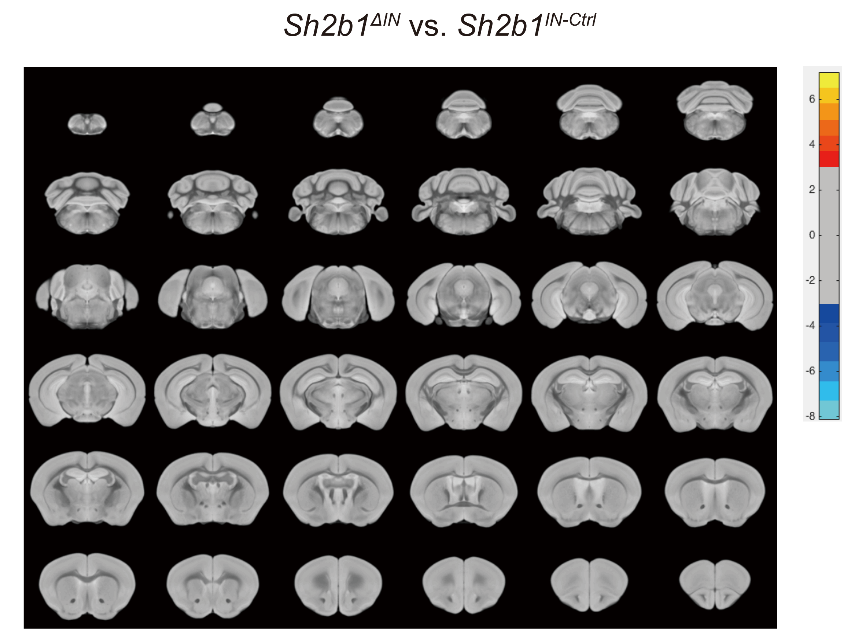


**Fig. S14. Effects of hippocampal interneuron-specific *Sh2b1* gene deletion on the brain anatomy.** The figure shows the results of voxel-based morphometry (VBM). The legend represents the *t*-value of the two-sample *t*-test. In the figure, red represents the area where the gray matter density of the experimental group significantly increased compared to the control group, while blue represents the area where the gray matter density of the experimental group significantly decreased compared to the control group. The clusters shown were corrected for false discovery rate (FDR) and *p* < 0.05. The viewing angle is the coronal view from the head to the tail side, so the actual left and right of the mice are opposite to the left and right shown in the image.


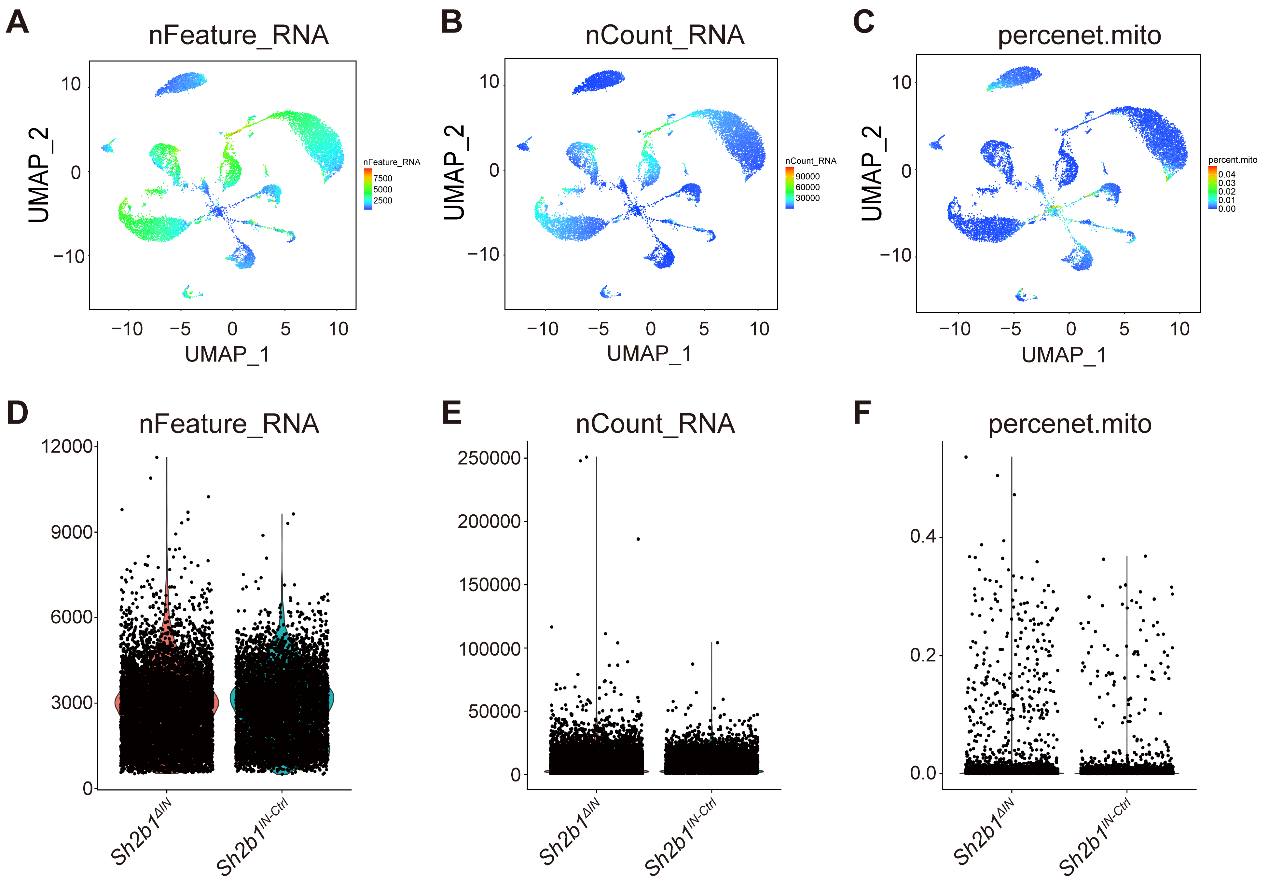


**Fig. S15. Data quality for single nuclei gene expression analysis.** Quality control UMAP plots showing RNA features (**A** and **D**), counts (**B** and **E**), and percentage of mitochondrial reads (**C** and **F**) per cell, respectively.


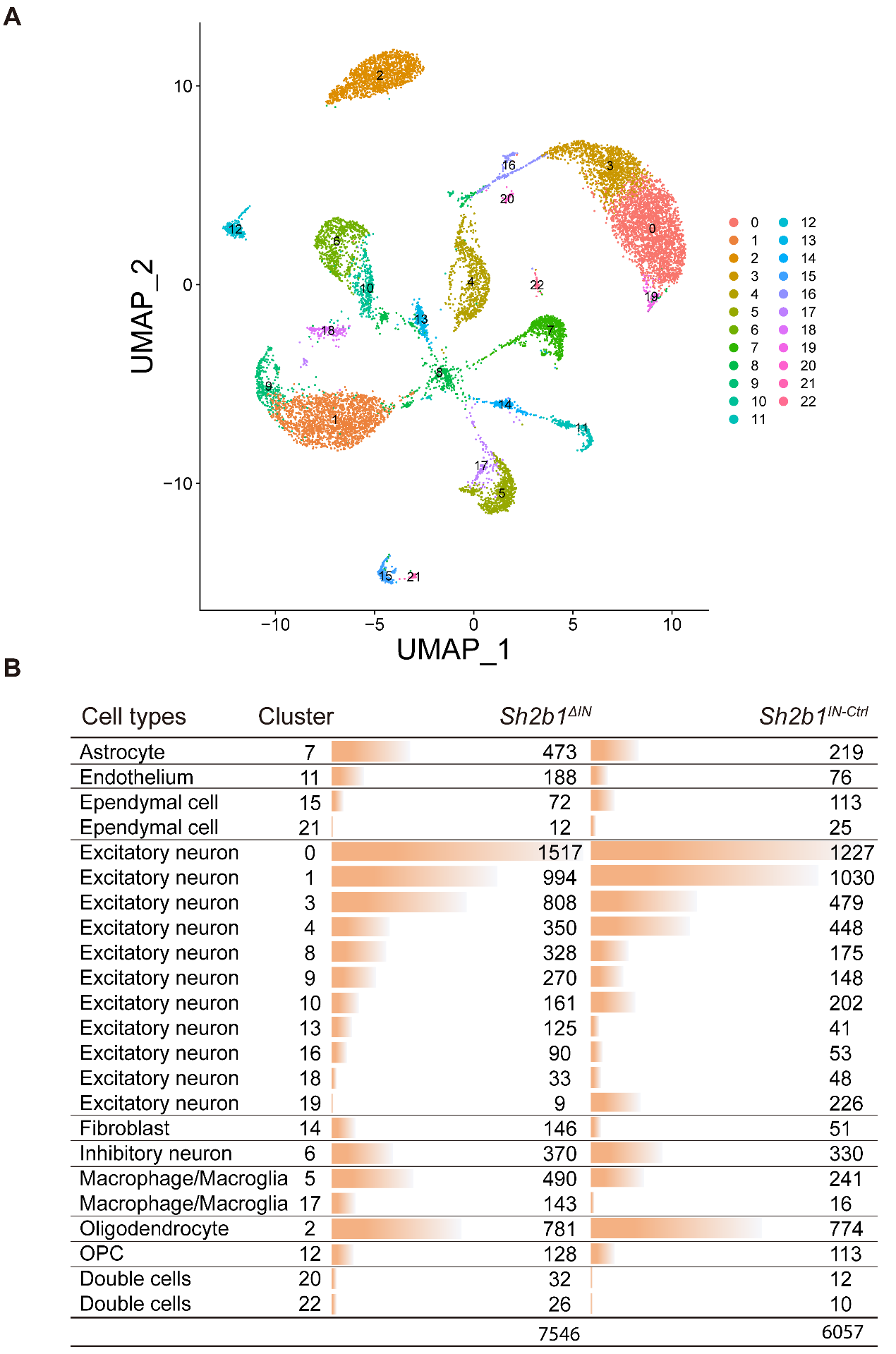


**Fig. S16. The cell clusters identified by 10x Genomics.** (**A**) UMAP plot of integrated datasets colored by 23 cell clusters, sampled. Dots, individual cells; colors, cell clusters. (**B**) Table shows the cell type and number of cells in each cell cluster in the hippocampus of *Sh2b1^ΔIN^* versus *Sh2b1^IN-Ctrl^* mice.


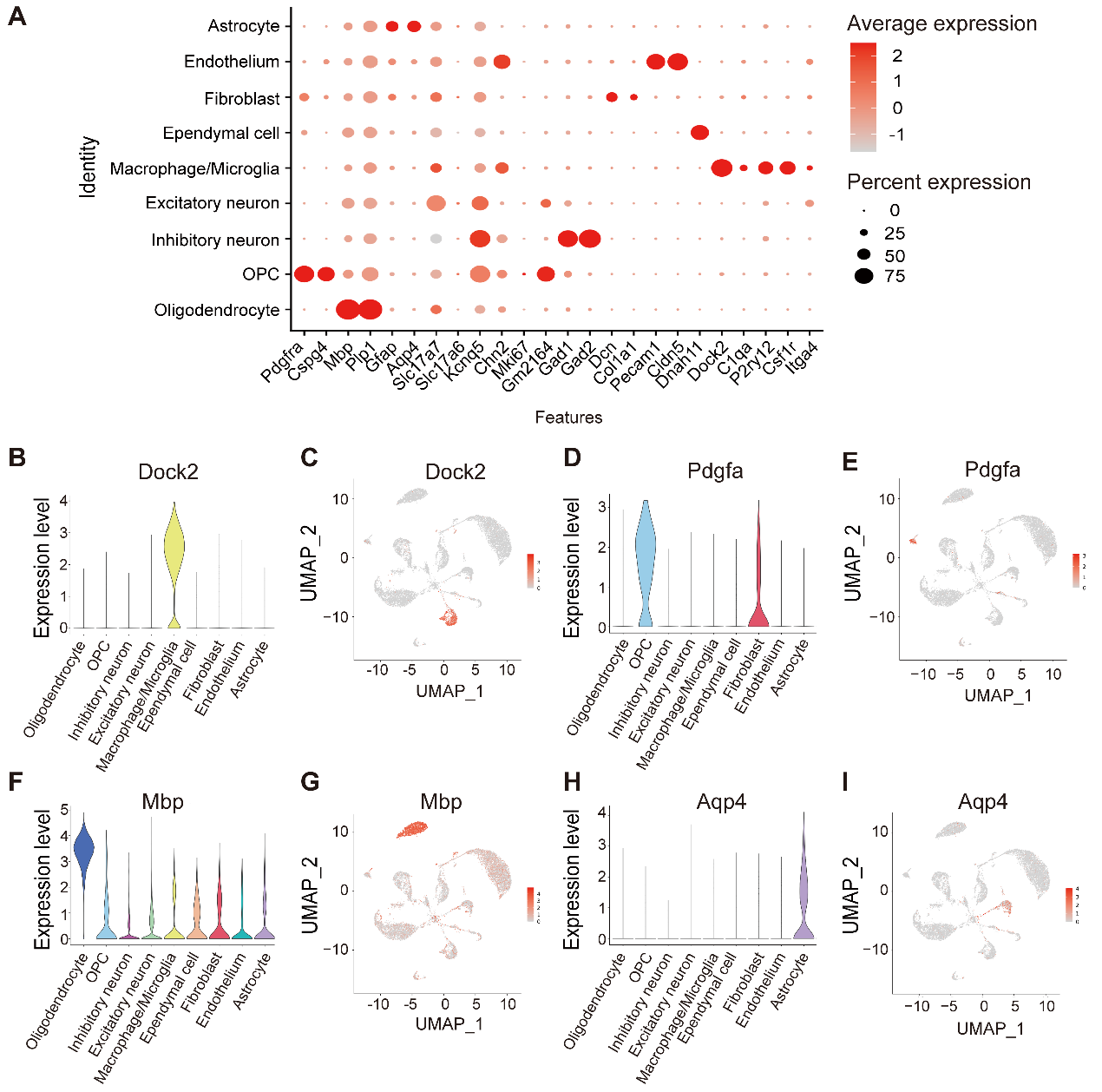


**Fig. S17. The cell clusters and marker genes identified by 10x Genomics.** (**A**) Dot plot shows the expression of key marker genes in each cell type clusters. The size of the dot indicates the percentage of cells expressing the gene in each cluster, and the color depicts the average normalized transcript counts in the cells. (**B**−**I**) Feature violin plot and UMAP plot show the cell type-specific marker genes *Dock2* (**B** and **C**), *Pdgfra* (**D** and **E**), *Mbp* (**F** and **G**), and *Aqp4* (**H** and **I**) in macrophage/microglia, oligodendrocyte precursor cell (OPC), oligodendrocyte, and astrocyte, receptively.


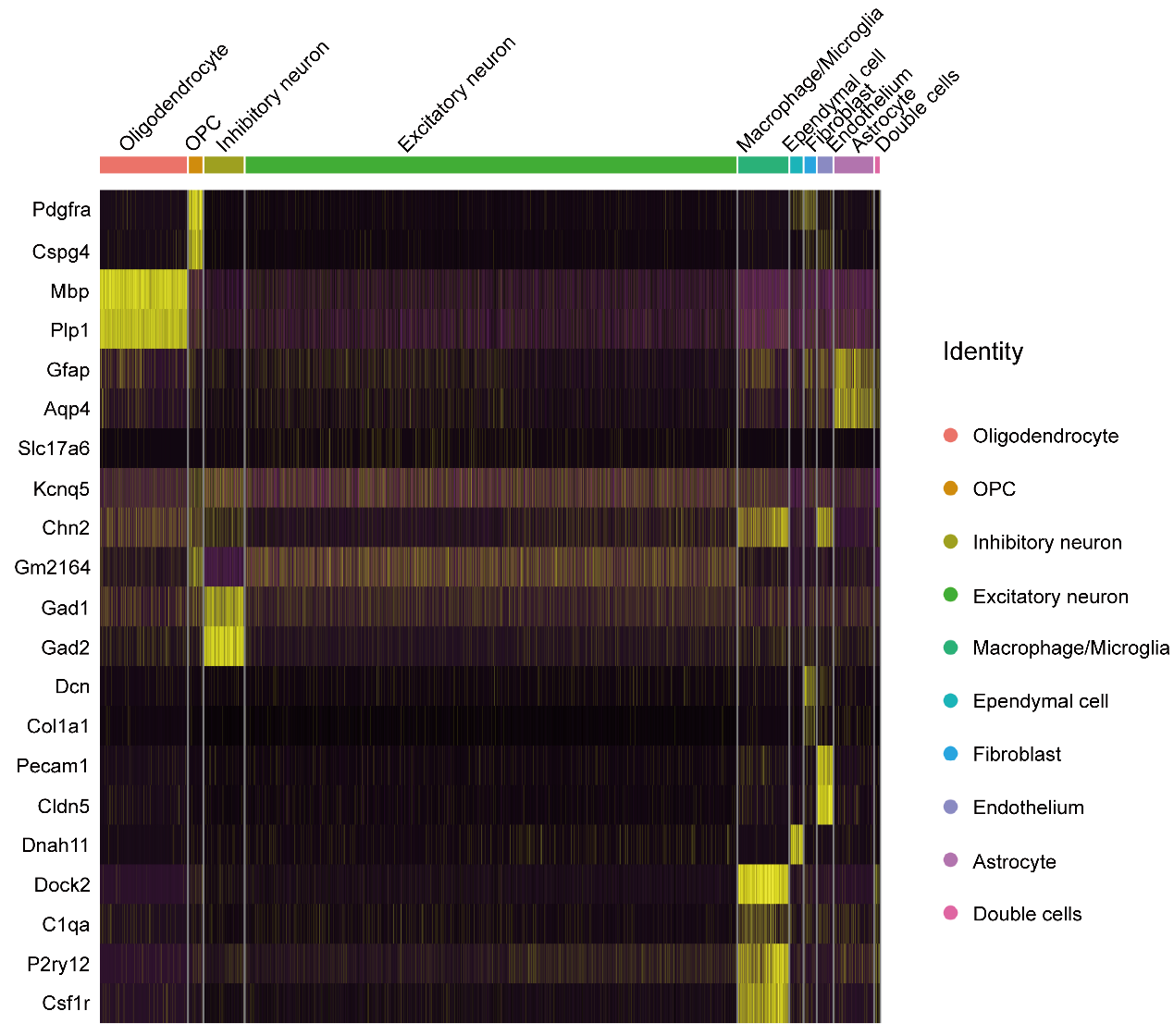


**Fig. S18. Heatmap showing expression of signature genes in representative cell types in multiple cell clusters.** The color represents the scaled transcript counts.


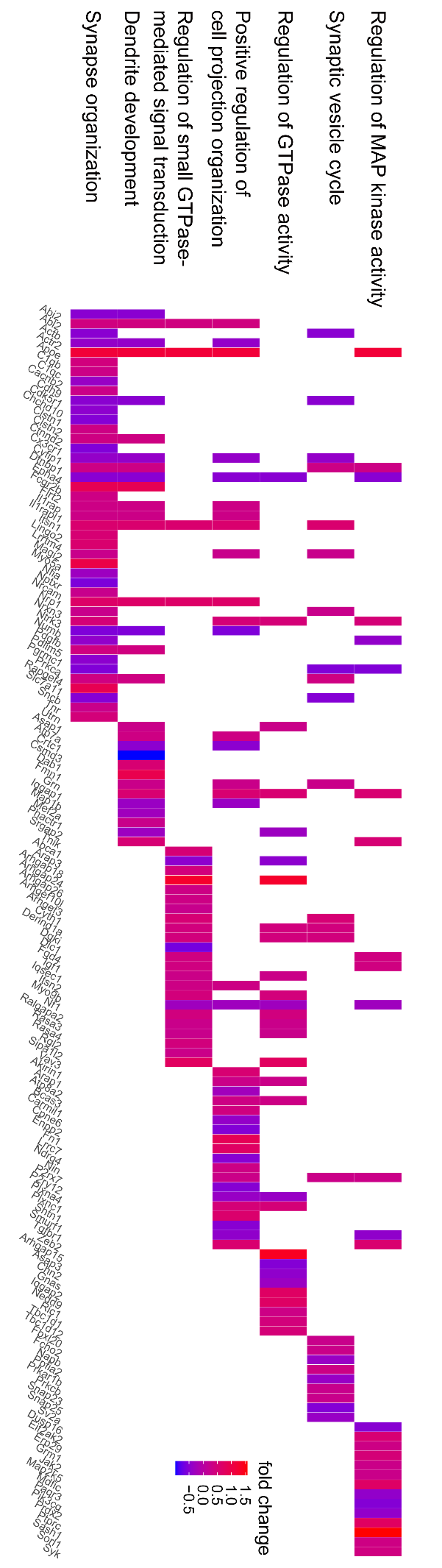


**Fig. S19. Top GO terms enriched for differentially expressed genes in the comparison of *Sh2b1^ΔIN^* versus *Sh2b1^IN-Ctrl^* hippocampal inhibitory neuronal nuclei.**


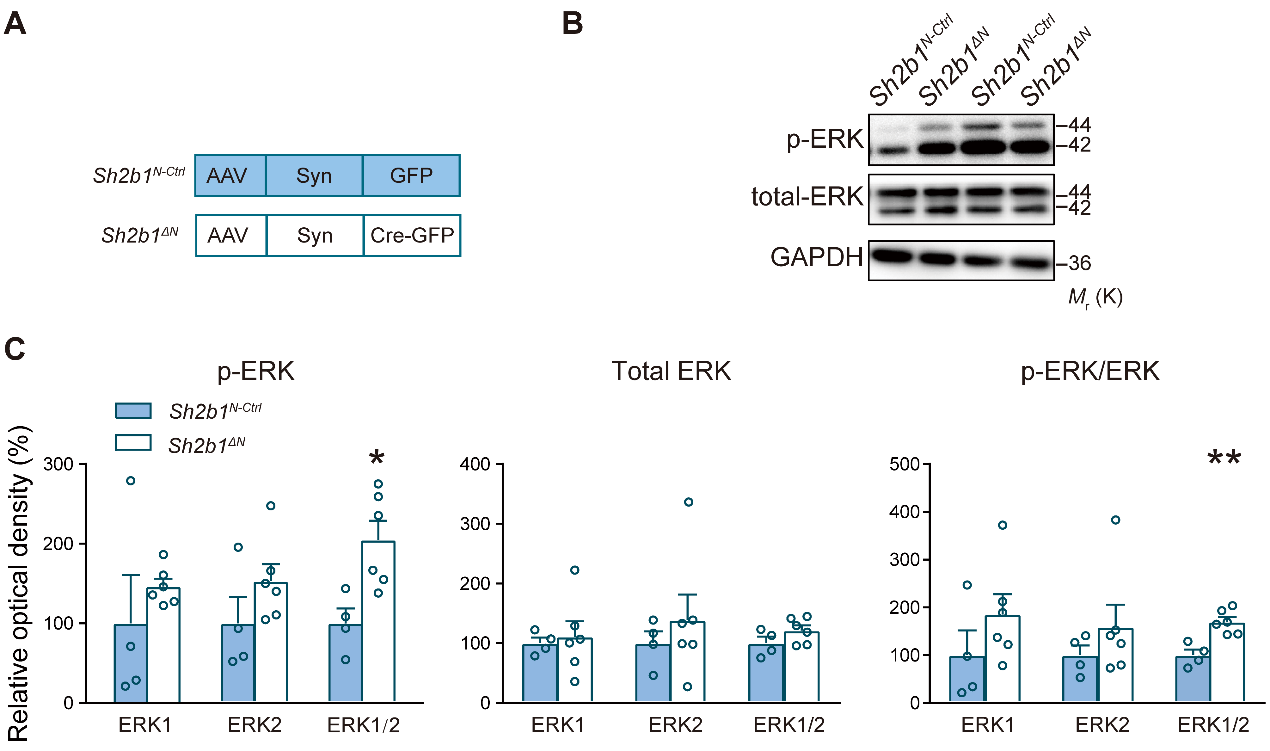


**Fig. S20. Effects of hippocampal *Sh2b1* deletion on ERK signaling.** (**A**) Schematics of AAV injections to obtain mice null for *Sh2b1* selectively in hippocampal neurons (*Sh2b1^ΔΝ^*, while *Sh2b1^Ν-Ctrl^* as the control group). (**B** and **C**), Representative immunoblots (**B**) and pooled data (**C**) show the phosphorylated (“p-”) and total protein abundance of ERK in the hippocampus of mice null for *Sh2b1* in hippocampal neurons. *Sh2b1^Ν-Ctrl^*, n = 4; *Sh2b1^ΔΝ^*, n = 6. *Left*, p-ERK, *p* = 0.3614, 0.1907, and 0.0136 (*), *Sh2b1^Ν-Ctrl^* versus *Sh2b1^ΔΝ^*, for ERK1, ERK2, and ERK1/2, respectively, unpaired Student’s *t*-test. *Middle*, total ERK, *p* = 0.7574, 0.5054, and 0.1658, *Sh2b1^Ν-Ctrl^* versus *Sh2b1^ΔΝ^*, for ERK1, ERK2, and ERK1/2, respectively, unpaired Student’s *t*-test. *Right*, the ratio of p-ERK and total ERK, *p* = 0.2369, 0.3583, and 0.0023 (**), *Sh2b1^Ν-Ctrl^* versus *Sh2b1^ΔΝ^*, for ERK1, ERK2, and ERK1/2, respectively, unpaired Student’s *t*-test.


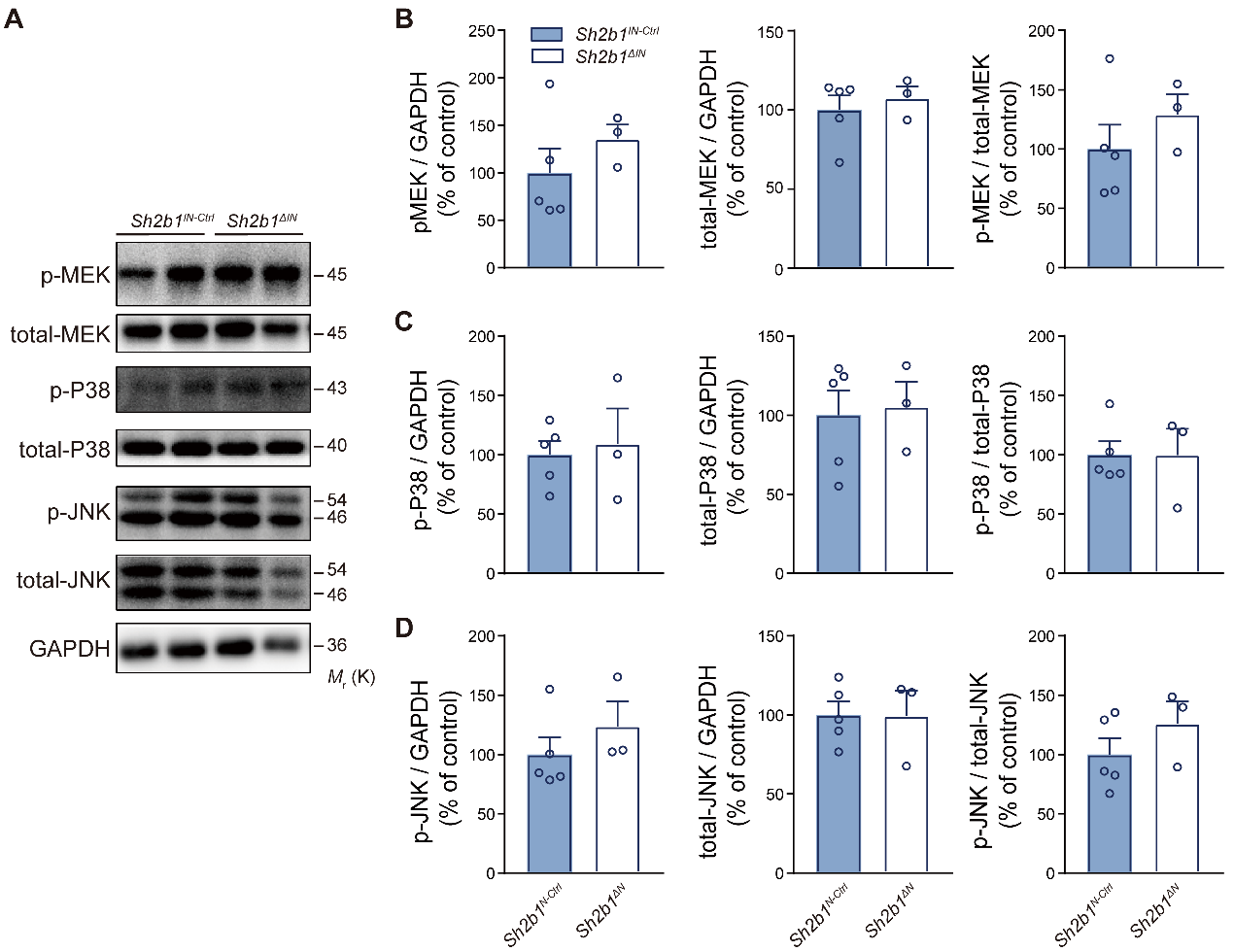


**Fig. S21. Effects of hippocampal *Sh2b1* deletion on MEK, P38, and JNK signaling.** (**A**) Representative immunoblots (**A**) and pooled data (**B**−**D**) show that protein expression in hippocampal CA1 regions. The immunoreactivity of phosphate protein was normalized to that of the total protein and is expressed as percentage of the control group. Data are shown as the mean ± S.E.M. *Sh2b1^IN-Ctrl^*, n = 5; *Sh2b1^ΔIΝ^*, n = 3. (**B**) *p* = 0.3579, 0.5899, and 0.3684, *Sh2b1^IΝ-Ctrl^* versus *Sh2b1^ΔIΝ^*, for p-MEK, total-MEK, and the ratio of p-MEK and total MEK, respectively, unpaired Student’s *t*-test. **(C**) *p* = 0.7453, 0.8309, and 0.9858, *Sh2b1^IΝ-Ctrl^* versus *Sh2b1^ΔIΝ^*, for p-P38, total-P38, and the ratio of p-P38 and total P38, respectively, unpaired Student’s *t*-test. (**D**) *p* = 0.3672, 0.9688, and 0.2946, *Sh2b1^IΝ-Ctrl^* versus *Sh2b1^ΔIΝ^*, for p-JNK, total-JNK, and the ratio of p-JNK and total JNK, respectively, unpaired Student’s *t*-test.
